# Exotic mechanical properties enabled by countersnapping instabilities

**DOI:** 10.1073/pnas.2423301122

**Published:** 2025-04-17

**Authors:** Paul Ducarme, Bart Weber, Martin van Hecke, Johannes T. B. Overvelde

**Affiliations:** ^a^Autonomous Matter and Infomatter Departments, AMOLF, Amsterdam 1098 XG, The Netherlands; ^b^Materials Department, Advanced Research Center for Nanolithography, Amsterdam 1098 XG, The Netherlands; ^c^Van der Waals-Zeeman Institute, Institute of Physics, University of Amsterdam, Amsterdam 1098 XH, The Netherlands; ^d^Huygens-Kamerlingh Onnes Lab, Leiden Institute of Physics, Universiteit Leiden, Leiden NL-2300 RA, The Netherlands; ^e^Department of Mechanical Engineering, Institute for Complex Molecular Systems, Eindhoven University of Technology, Eindhoven 5600 MB, The Netherlands

**Keywords:** snapping, programmability, geometric nonlinearities, elastic instabilities, mechanical metamaterials

## Abstract

Mechanical snapping instabilities are leveraged by natural systems, metamaterials, and devices for rapid sensing, actuation, and shape changes, as well as to absorb impact. In all current forms of snapping, shapes deform in the same direction as the exerted forces, even though there is no physical law that dictates this. Here, we realize countersnapping mechanical structures that respond in the opposite way. In contrast to regular snapping, countersnapping manifests itself in a sudden shortening transition under increasing tension or a sudden increase in tensile force under increasing extension. We design these structures by combining basic flexible building blocks that leverage geometric nonlinearities. We demonstrate experimentally that countersnapping can be employed to obtain new exotic properties, such as unidirectional stick–slip motion, switchable stiffness that does not otherwise affect the state of the system, and passive resonance avoidance. Moreover, we demonstrate that combining multiple countersnapping elements allows sequential stiffness switching for elements coupled in parallel, or instantaneous collective switching for elements in series. By expanding the repertoire of realizable elastic instabilities, our work opens routes to principles for mechanical sensing, computation, and actuation.

## Significance

From an umbrella flipping inside out during a gust of wind to a slender stick bowing when compressed, mechanical instabilities are often seen as undesirable. However, they can also be leveraged, as illustrated by the snapping-based prey capture strategies of the Venus flytrap and mantis shrimp. Inspired by these observations, researchers have started to harness such nonlinear effects to design materials with exotic and programmable functions. Here, we expand this repertoire by experimentally demonstrating countersnapping, where a combination of geometrically nonlinear building blocks cooperate to suddenly contract when increasingly tensioned. We demonstrate that this behavior unlocks exotic mechanical and dynamical behavior, potentially useful for metamaterials, sensors, and smart structures.

**Fig. 1.**
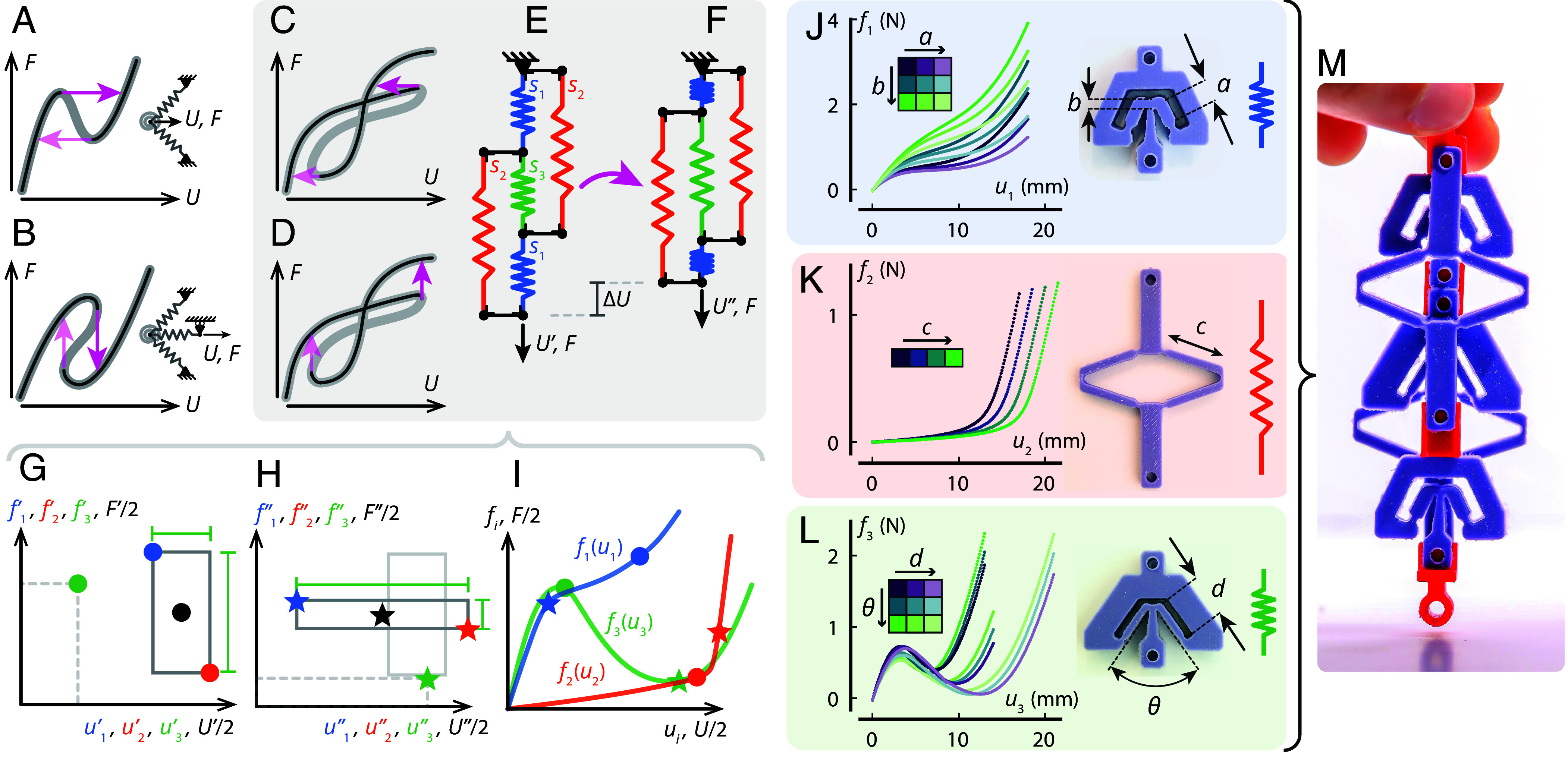
Combining nonlinear mechanical building blocks to realize countersnapping instabilities. Regular snapping instabilities observed in (*A*) a nonmonotonic force (*F*)–displacement (*U*) relation realized by a pair of springs forming a left buckled beam and driven from the connection point, and (*B*) a multivalued force–displacement relation achieved by serially coupling a nonmonotonic element to a spring. (*C* and *D*) Self-intersecting force–displacement relation that leads to countersnapping: (*C*) a sudden decrease in *U* when *F* is increased, or (*D*) a sudden increase in *F* when *U* is increased. (*E* and *F*) Realization of countersnapping elements with a network of three different weakly nonlinear springs ($s_{1}-s_{3}$). (*G*–*I*) Geometric construction of the force–displacement curves needed to achieve the countersnapping behavior. The color of the markers refer to the type of spring. Black markers refers to the global state. In (*G* and *H*), the dark gray rectangles represent the state before (*G*) and after (*H*) switching. The width and height of each rectangle are used to create the green marker which represents the state of spring *s*_3_. In (*I*), markers from both (*G* and *H*) are plotted together, which allows to draw the three local force–displacement curves. (*J*–*L*) Experimentally evaluated nonlinear building blocks for a range of parameters. (*J*) Softening building block with a=[4,5,6] mm, b=[1.0,1.5,2.0] mm. (*K*) Stiffening building block with c=[12,13,14,15] mm. (*L*) Nonmonotonic building block with d=[5,7,9] mm, $\theta =[50^{{}^{\circ}},60^{{}^{\circ}},70^{{}^{\circ}}]$. (*M*) Assembly of the building blocks that lead to countersnapping.

Historically, structural stability has been a strong requirement for the design of mechanical systems. In recent years, however, a paradigm shift toward embracing and harnessing instabilities has enabled materials and structures that embody complex functionalities. Examples can be found in fields as diverse as mechanical metamaterials (1–4), signal propagation (5–7), shape-morphing structures (8–10), deployable structures (11), surface patterning (12, 13), mechanical computation (14–16), and soft robotics (17–22). Snapping instabilities, the sudden transition between two distinct configurations (Fig. 1 *A* and *B*), have demonstrated to be a fundamental building block for these applications.

Snapping is often associated with a nonmonotonic force–displacement curve, which leads to discontinuous changes in displacement triggered by an incremental change in force (Fig. 1*A*). Additionally, a multivalued force–displacement curve can lead to discontinuous changes in force under incremental displacements (Fig. 1*B*). Based on these characteristics, snapping instabilities can be leveraged to amplify small input loads into large deformations (19, 23), to endow soft robots with fast actuation and jumping capabilities (20, 22, 24), to realize metamaterials with unprecedented shock-absorbing properties (2), to passively convert a steady stream of energy into functional pulsatile outputs (17, 25) or to accurately morph structures into various stable shapes (8–10, 26, 27). Structures built from multiple snapping elements display rich deformation pathways characterized by a succession of snapping events (28–32), which has been utilized to generate locomotion gaits for pneumatic soft robots (33) and deterministic deployment sequences (11, 34). Snapping transitions and multistability have also proven to be pivotal ingredients to store and process information in mechanical platforms (14–16).

Still, all these exotic properties arise from snapping events that have one aspect in common: snapping always occurs in the same direction as the incremental load. For example, under controlled force, pulling leads to a sudden extension, and under controlled deformation, stretching leads to a sudden force drop (Fig. 1 *A* and *B*). While this link between the direction of forces and deformations seems natural, there is no physical law that requires it. Theoretical work has shown that it should be possible to observe what we refer to as countersnapping, where pulling leads to a sudden contraction and incremental stretching leads to a sudden and sharp increase in force (35). Yet, it is an open question how to design and experimentally realize a (meta)structure that exhibits this behavior, with only limited experimental work hinting toward—but not demonstrating—the existence of such counterintuitive snapping response (36), and no reported occurrence in natural systems. Importantly, it remains an open question what kind of mechanical exotic—yet useful—behavior can be realized by leveraging such countersnapping instabilities.

Here, we design, fabricate, and investigate structures that materialize such countersnapping behavior. Our structure employs three different building blocks, each with a different type of nonlinear geometric behavior, and combines these in a small network that collectively realizes a self-intersecting force–displacement relation. Moving along such an equilibrium path by either driving the force or displacement produces countersnapping (Fig. 1 *C* and *D*). Using this physical implementation, we show how countersnapping manifests itself under various loading conditions and experimentally demonstrate unique properties originating from the self-intersection observed in the force–displacement curve. We show unidirectional stick–slip motion under cyclic driving, programmable stiffness that does not modify the externally observed state of the structure, self-switching stiffness for resonance avoidance, and imperfection-insensitive collective snapping sequences at constant deformation.

## Materializing Countersnapping Instabilities by Coupling Nonlinear Mechanical Building Blocks

Countersnapping instabilities can be seen as a mechanical counterpart of Braess’ paradox (35). In its original formulation, this paradox describes how closing a road can improve traffic flow (37). Similarly, one can construct a set of springs connected by strings, such that under a constant tensile load, the cutting of a taut string leads to a contraction of the whole assembly (38, 39). The essence of this construction is multistability, where an initial pretensioned “serial” configuration of springs transforms into a “parallel” configuration and where the trigger for this transition is the removal of a single link. More generally, such behavior can occur in systems with sufficiently complex energy potentials, as was shown theoretically using a set of particles interacting through strongly nonlinear potentials, and also using a set of linear springs that exhibit geometric nonlinearities under tension, originating from changes in orientation (35). However, the former design requires steep potentials (e.g., Lennard-Jones potential), whereas the latter design requires 21 perfectly hinging springs with required stiffnesses that vary over 6 orders of magnitude. Experimentally, overcoming these design requirements has proven prohibitively hard, with a recent attempt only hinting toward the existence of countersnapping as the experiments were dominated by viscoelastic behavior (36). Hence, while inspiring, it remains an open question how to robustly realize countersnapping instabilities experimentally.

### Construction of Local Force–Displacement Curves.

To realize reversible countersnapping behavior, we focus on tensile deformation and modify the spring-network geometry that exhibits the mechanical pendant of Braess’ paradox (35). Note that this spring network is not proven to be the simplest countersnapping mechanical system, yet it constitutes a useful starting point for the rational design that we are presenting here.

We assume that its five elastic springs are nonlinear and of three distinct types *s*_1_, *s*_2_, and *s*_3_. Through symmetry, springs of type *s*_1_ and *s*_2_ occur twice in the network, yet we assume that they are in the same state (Fig. 1 *E* and *F*). At any point during the loading process, a spring *s*_*i*_ has a certain elongation noted *u*_*i*_ and a certain tensile force noted *f*_*i*_.

Our design target is to construct local force–displacement $f_{i}\left(u_{i}\right)$ curves for $s_{1}-s_{3}$, such that, for a given global force *F*, the network has two stable configurations with global extensions $U^{\prime }$ and $U^{\prime \prime }$. Essential is that the system switches from $U^{\prime }$ to $U^{\prime \prime }$ at an incremental increase in force, with $U^{\prime \prime }<U^{\prime }$. To construct the local curves $f_{i}\left(u_{i}\right)$, we develop a method that relies on a geometric interpretation of the constraints and force balance equations that govern the network shown in Fig. 1*E*. From geometry and force balance, we indeed find that the global displacement is the sum of the extensions of springs *s*_1_ and *s*_2_ ($U=u_{1}+u_{2}$), and the global force is the sum of the tensions in springs *s*_1_ and *s*_2_ ($F=f_{1}+f_{2}$). Additionally, the extension of spring *s*_3_ is the difference in extension between springs *s*_2_ and *s*_1_ ($u_{3}=u_{2}-u_{1}$), and its tension is the difference in tension between springs *s*_1_ ad *s*_2_ ($f_{3}=f_{1}-f_{2}$). By using these relations, the state of the structure can be geometrically represented by drawing a rectangle in a force–displacement plane (Fig. 1 *G* and *H*). The *Top-Left* corner of the rectangle coordinates represents the stretch *u*_1_ and tension *f*_1_ in spring *s*_1_, while the *Bottom-Right* corner coordinates represent those in *s*_2_. Since $u_{3}=u_{2}-u_{1}$ and $f_{3}=f_{1}-f_{2}$, the width and height of that rectangle are the stretch *u*_3_ and tension *f*_3_ in spring *s*_3_. Moreover, since $U/2=(u_{1}+u_{2})/2$ and $F/2=(f_{1}+f_{2})/2$, the coordinates of the rectangle’s center point represent half the global displacement U/2 and half the global force F/2.

In Fig. 1*G*, a first rectangle is drawn to represent the state before switching (Fig. 1*E*), while in Fig. 1*H*, a second rectangle is drawn to represent the state just after switching (Fig. 1*F*). Crucially, the center of the second rectangle must be shifted horizontally to the left compared to the center of the first rectangle (as $U^{\prime \prime }<U^{\prime }$ and the force *F* is conserved). Next, in Fig. 1*I*, we plot together the *Top* and *Bottom* corners of both rectangles, along with two extra markers whose coordinates are the width and height of both rectangles (green dot, green star). Finally, for each of the three springs, a force–displacement curve is drawn through the origin and corresponding markers. To ensure that the first state becomes unstable for an infinitesimal increase in *F*, the curve $f_{3}\left(u_{3}\right)$ must meet additional conditions detailed in the appendix (*SI Appendix*, section 2). To maintain monotonic force–displacement curves for *s*_1_ and *s*_2_, we can retrospectively notice that the second rectangle (Fig. 1*H*) must be wider and flatter than the first one, indicating that *s*_3_ has a higher stretch and a lower tension after the switch, similar to the cut spring in the Braess’ paradox analog (38, 39).

Most importantly, this geometric construction reveals that the force–displacement response of *s*_1_ needs to be softening, *s*_2_ stiffening, and *s*_3_ nonmonotonic (Fig. 1*I*). Note that the same geometric construction can also be applied to identify the individual curves leading to countersnapping during unloading, that is a sudden increase in elongation as the tensile force is slowly reduced (*SI Appendix*, section 3 and Fig. S4). A key insight unveiled by this approach is that a countersnapping instability is possible by using only three different weakly nonlinear potentials, which can be materialized using relatively basic elastic building blocks as we will demonstrate next.

### Materializing Nonlinear Building Blocks.

Our approach to realize the countersnapping behavior in experiments requires designing and fabricating several monolithic and elastic structures, each materializing one of the three types of nonlinear springs. These monolithic structures will then be assembled together in the arrangement shown in Fig. 1*E*. However, designing manufacturable structures with exact force-extension curves is a difficult inverse design problem (40, 41). Instead, we focus on building an experimental library of building blocks that demonstrate the general profile of the desired softening, stiffening, and nonmonotonic force-extension behavior and then evaluating assemblies of these nonlinear springs numerically. We focus on using geometric nonlinearities, but other approaches where material nonlinearities or contact are harnessed could potentially be used. For both the softening and nonmonotonic building block designs (Fig. 1 *J* and *L*), we use a v-shaped pair of inclined beams, wedged within a relatively stiffer structure (8, 26). The stiffening building block design (Fig. 1*K*) is based on a diamond-shaped set of beams connected by flexures that, upon tension, transition from a soft-and-bending to a stiff-and-stretching mode of deformation (*SI Appendix*, section 1A and Fig. S1 *A*–*C* for detailed drawings).

Next, we fabricated several centimeter-scale samples of the three different building blocks, each spanning a parameter range (Fig. 1*J*–*L*). The structures are fabricated by casting a silicone (Smooth-Sil 945, Smooth-On) in a 3d-printed mold (PolyJet Eden260VS, Stratasys) (*SI Appendix*, section 1A and Fig. S1). We then measured the force-extension response (Instron model 5965, 100-N load cell) of all the structures individually. In total, our catalog of building blocks with different nonlinear behaviors consists of nine units for the softening, four units for the stiffening, and nine units for the nonmonotonic behavior. We then developed a numerical algorithm to simulate the behavior of assemblies composed of these building blocks, and assess whether they display a countersnapping instability (*SI Appendix*, section 1B). By running all possible combinations in our catalog (324 simulations), we found eight potentially countersnapping assemblies (*SI Appendix*, Fig. S2*E*). This low percentage suggests that the countersnapping effect is relatively sensitive to the geometric parameters. A more detailed study of the influence of the geometric parameters reveals that the countersnapping effect exists in a relatively narrow yet feasible window of geometric parameters (*SI Appendix*, section 4 and Fig. S5).

### Experimental Observation of a Countersnapping Instability.

**Fig. 2.**
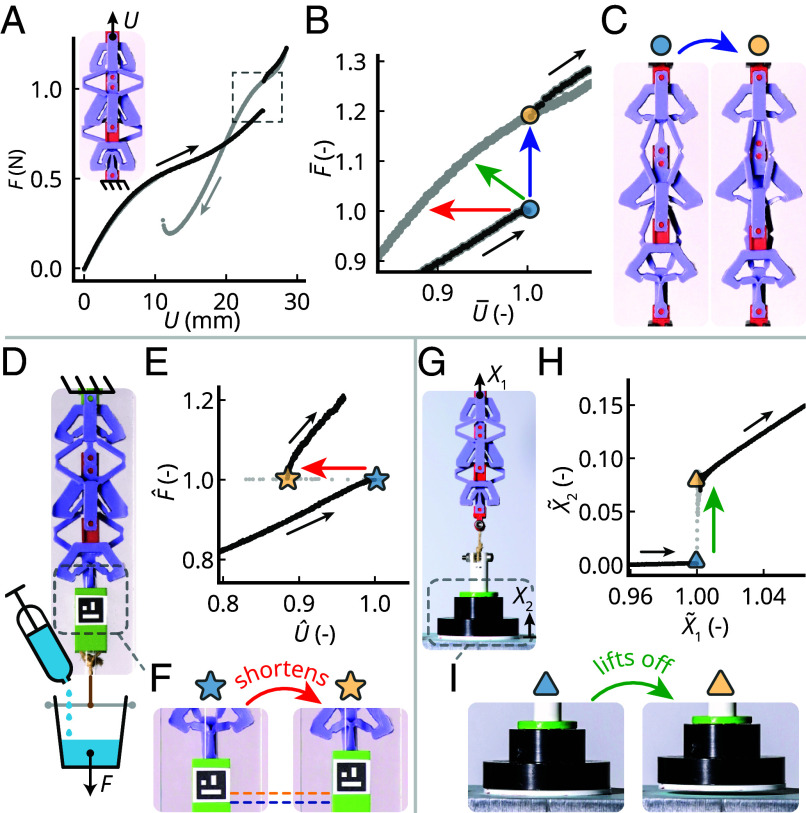
Experimental observation of countersnapping behavior. (*A*) Force–displacement curve for a countersnapping assembly obtained by increasing (dark gray) then decreasing (light gray) the applied displacement *U* and measuring the reaction force *F*. (*B*) Close-up on the critical point indicated in (*A*). The axes have been normalized $\overset{¯}{U}=U/U_{\text{c}}$, $\overset{¯}{F}=F/F_{\text{c}}$, where the subscript c indicates the critical point under controlled displacement. $U_{\text{c'}}=25.2$ mm, $F_{\text{c'}}=0.88$ N. Colored arrows indicate the jumps under controlled displacement (blue), controlled force (red), and mixed conditions (green). (*C*) Snapshots of the configuration just before (*Left*) and just after (*Right*) snapping during loading (displacement-controlled) (*D*) Setup for the force-controlled experiment: The structure is attached from the *Top* and a cup, suspended at the *Bottom*, is slowly filled with water. (*E*) Force–displacement curve obtained by increasing the applied force *F* and measuring the displacement *U*. The axes have been normalized $\hat{U}=U/U_{\text{c'}}$, $\hat{F}=F/F_{\text{c'}}$, where the subscript c’ indicates the critical point under controlled force. $U_{\text{c'}}=23.9$ mm, $F_{\text{c'}}=0.81$ N. (*F*) Snapshots just before (*Left*) and just after (*Right*) snapping. (*G*) Setup for loading under mixed conditions, measuring the elevation *X*_2_ of the weight. The structure is pulled upward from the *Top* by an increasing displacement *X*_1_ while the weight attached to the *Bottom* is initially sitting on a flat platform. (*H*) Weight elevation as a function of the applied displacement. The axes have been normalized ${\tilde{X}}_{1}=X_{1}/X_{1\;\text{c''}}$, ${\tilde{X}}_{2}=X_{2}/X_{1\;\text{c''}}$, where the subscript c” indicates the critical point under mixed conditions. $X_{1\;\text{c''}}=23.5$ mm. (*I*) Snapshots just before (*Left*) and just after (*Right*) the sudden liftoff. Notice the higher weight elevation in the *Right* snapshot.

To observe the countersnapping behavior experimentally, we physically assemble the five building blocks of one of the eight potential assemblies (Fig. 1*M* and *SI Appendix*, Figs. S2*F* and S3) and perform three mechanical tests (Fig. 2). First, we characterize the force-extension behavior of the nonlinear spring network under controlled extension using a tensile tester. We observe that when the extension *U* reaches a critical value, the element snaps and the reaction force suddenly jumps to a higher value—a first and clear signature of countersnapping behavior (Fig. 2*A*–*C* and Movie S1). Note that during unloading, we observe an ordinary snapping event, where this asymmetry is the result of the intersection in the force–displacement behavior. Based on energy considerations, we argue that an elastic system that would countersnap upon both loading and unloading should be possible but would necessitate a more complex force–displacement curve with two points where stable branches intersect (*SI Appendix*, section 5 and Fig. S6).

Second, we study countersnapping under constant load. To do so, we suspend the structure and load it by slowly filling a cup attached to one side of the structure with water, while measuring the force and extension using a load cell and video tracking. Once the critical tensile load is reached, we observe that the structure suddenly reduces its elongation by about 12%, leading to an upward jump of the suspended cup—a second hallmark of countersnapping (Fig. 2*D*–*F* and Movie S2).

Third, we demonstrate that countersnapping also can lead to a simultaneous discontinuous contraction and force increase, by slowly lifting an object that initially is resting on a surface (Fig. 2*G*). For an appropriate weight that lies between the forces observed before and after snapping in the controlled displacement experiment, increasing the lifting force triggers the countersnapping instability and leads to a sudden lifting of the suspended weight (Fig. 2*G*–*I* and Movie S3). Hence, depending on the loading conditions, the countersnapping spring network exhibits a combination of a sudden increase in force and contraction (Fig. 2*B*), which originates from the switch from a predominantly series to a predominantly parallel distribution of the load (*SI Appendix*, section 6 and Fig. S7).

## Exotic Properties Enabled by Countersnapping

Having demonstrated experimentally the existence of the countersnapping instability, we next explore the functionalities of a single countersnapping element. First, we harness its unusual combination of snapping directions under cyclic loading; then we exploit the functionalities originating from the self-intersection of the force–displacement curve.

### Unidirectional Stick–Slip Actuation.

Stick–slip actuation is an important mechanism for precision manipulation and positioning applications (42, 43). It relies on a combination of an external actuator and a frictionally coupled slider, so that alternating slow and fast actuation produces sticking and incremental slipping between slider and actuator. Snapping could in principle be used to generate the fast phase of actuation, reducing the need for intricate control systems. However, as ordinary snapping occurs in the direction of the applied force (Fig. 1*A*), cyclic actuation drives snapping events in opposite directions with little net effect. By contrast, our countersnapping element snaps in the same direction during loading and unloading, resulting in two successive contractions per cycle (Fig. 1*C*), which enables unidirectional stick–slip actuation under cyclic loading.

**Fig. 3.**
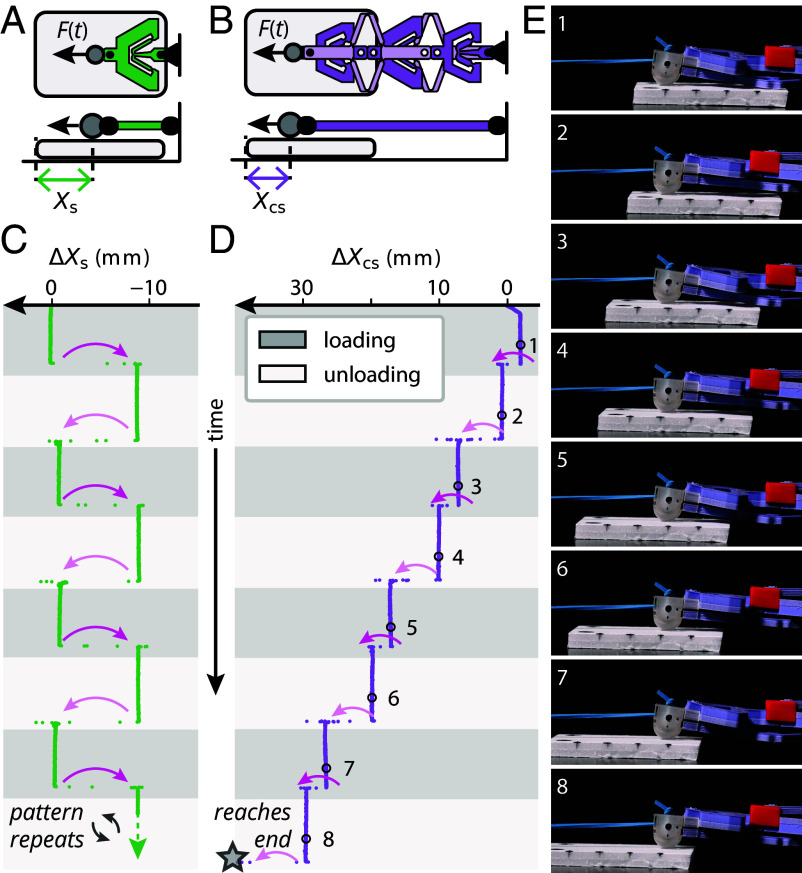
Experimental observation of unidirectional stick–slip behavior. (*A* and *B*) Sliding experiment setup used for the snapping (*A*) and countersnapping (*B*) structures. The block of foam is depicted by the gray rounded rectangles. (*C* and *D*) Change in distance between the contact point and the piece of foam over time, for the (*C*) snapping and (*D*) countersnapping structures. The gray star indicates the instant at which the countersnapping structure completely slides off the piece of foam. Each loading and unloading sequence lasts 9.38 s (±1.9%) in (*C*) and 16.87 s (±0.4%) in (*D*). (*E*) Snapshots of the sliding experiment using the countersnapping structure (side view) taken at fixed time intervals.

To demonstrate this, we study the behavior of elastic structures in the following setup. We horizontally suspend the structure, clamp its right side, and rest its left side on a 5 cm-long block of foam placed on a rigid surface. We cyclically load its left side through a much softer elastic rubber band attached to a robotic arm. We compare the resulting motion of the foam block for an ordinary snapping spring (Fig. 3*A*) and our countersnapping structure (Fig. 3*B*). Note that the friction forces between the left side, foam, and surface are such that under smooth motion of the total structure, the foam block smoothly slides over the surface, and there is no sliding between the foam block and the elastic structure. However, when snapping instabilities are triggered, the structure and the foam exhibit relative sliding, as the inertial force caused by the large acceleration exceeds the static friction limit. As expected, for ordinary snapping, these events are of opposite sign, such that the total motion of the foam after one cycle is negligible (Fig. 3*C* and Movie S4). Instead, for our countersnapping element, the sliding motion is in the same direction during snapping and unsnapping, leading to a unidirectional motion of the foam block (Fig. 3 *D* and *E* and Movie S4). Hence, cyclic loading of a countersnapping element can be leveraged for unidirectional, incremental stick–slip motion, with potential applications in sensors that count numbers of loading cycles (44), stick–slip actuators (43), and soft robotic locomotion.

### Programmable Stiffness.

**Fig. 4.**
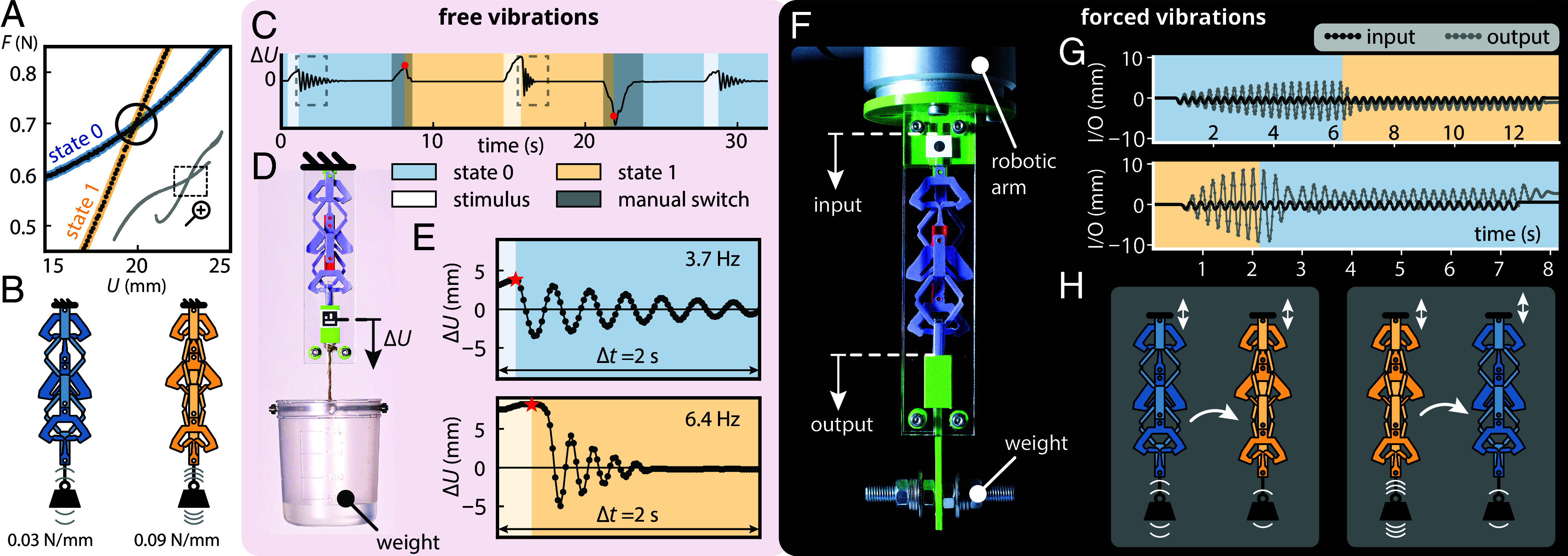
Experiments to demonstrate programmable and self-switching stiffness. (*A*) Close-up on the self-intersection of the force–displacement curve from Fig. 2*A*. (*B*) The two states, soft (0, blue) and stiff (1, yellow) that correspond to the intersection point in (*A*) have the same elongation under the same applied force, yet different stiffnesses. (*C*) Change in elongation ΔU over time during three episode of free oscillations. Each episode is triggered by a stimulus (slight pull and release) indicated by the whitened backgrounds. Between each episode, the state is manually switched by pulling or pushing on the weight, indicated by the darkened background. The red circles indicate the instant at which the state changes. The amplitude ΔU required to switch from 0 to 1 (resp. 1 to 0) is 5.0 mm (resp. −11.5 mm). (*D*) Experimental setup to for the free vibrations. (*E*) Close-up on the first two episodes of free oscillations highlighted by the boxes in (*C*). Each episode is triggered by a stimulus consisting of releasing the weight from a slightly stretched configuration compared to the intersection state, indicated by the red stars. The amplitude of that stretch is, respectively, 3.9 mm (*Top*) and 8.2 mm (*Bottom*). After releasing the weight when in state 0 (resp. state 1), the system oscillates freely at a frequency of 3.7 Hz (resp. 6.4 Hz). (*F*) Experimental setup for the forced vibrations. The sample is shaken vertically from the *Top* using a robotic arm (input), while the vertical position of the suspended weight is measured (output). (*G*) Evolution of the input and output displacements during forced vibrations, leading to resonance and a switch from soft to stiff (0→1, *Top*) and stiff to soft (1→0, *Bottom*). (*H*) Schematic representation of the resonance switches for the soft-to-stiff (0→1, *Left*) and stiff-to-soft (1→0, *Bottom*) transitions.

Our countersnapping element features an unusual point where the force–displacement curve self-intersects (Fig. 4*A*). Corresponding to this point are two distinct configurations characterized by the same extension and the same tensile force—yet by different stiffnesses (Fig. 4*B*). By measuring the slope of each branch at the intersection in the force–displacement plane, we characterized the stiffness of state 0 to be 0.03 N/mm (soft state), and of state 1 to be 0.09 N/mm (stiff state).

We now demonstrate that switching from one stiffness to the other can be achieved by means of snapping transitions and that this can be used to manipulate the oscillation frequency of a suspended mass, without affecting the elongation at equilibrium (Fig. 4*C*).

First, we load the countersnapping structure to the intersection point by suspending a cup of water, which applies a tensile dead load of around 0.7 N, resulting in an elongation of around 20 mm (Fig. 4*D*). To characterize the natural frequency, we excite the structure with a slight pull and release, while making sure that our element remains in its serial configuration (Fig. 4*B*, *Left*), and observe that the mass’ frequency equals 3.7 Hz (Fig. 4*E*, *Top* and Movie S5). Once the oscillations have damped out, we again pull on the suspended weight, but this time, up to the point where the structure snaps and switches to its parallel configuration (Fig. 4*B*, *Right*). By gently releasing the mass, the structure reaches the same equilibrium elongation, yet remains in the parallel configuration. When we now excite the structure by slightly pulling and releasing the mass, we observe a significantly higher frequency of 6.4 Hz (Fig. 4*E*, *Bottom* and Movie S5). This illustrates that countersnapping structures can be used to program the natural frequency of a mass-spring system—without changing the equilibrium load or extension.

This programmable stiffness allows the realization of passive self-switching of natural frequency for an externally driven mass-spring system. To demonstrate this, we suspend the countersnapping element from a robotic arm that allows vertical excitation at a specified frequency and attach a mass that stretches the countersnapping structure to its intersection point (Fig. 4*F*). Starting from the serial state, we vertically drive the robotic arm close to the resonance frequency. The effective vibrational live load results in oscillations that eventually trigger the countersnapping instability, causing the element to switch to its stiffer parallel state, which subsequently reduces the amplitude of the oscillations (Fig. 4*G*, *Top* and *H*, *Left* and Movie S6). Similarly, when the countersnapping element is initially in the parallel state and we set the driving frequency close to the corresponding natural frequency, the system eventually switches to the softer serial state, again leading to diminishing of the vibrations (Fig. 4*G*, *Bottom* and *H*, *Right* and Movie S6). Passively switching the natural frequency has minimum impact on the equilibrium deformation under the dead load, which stands in stark contrast with regular snapping structures actuated by resonance (45). Hence, countersnapping can be used to passively protect a system from specific vibrational resonances.

## Collective Behavior of Countersnapping Metamaterials

**Fig. 5.**
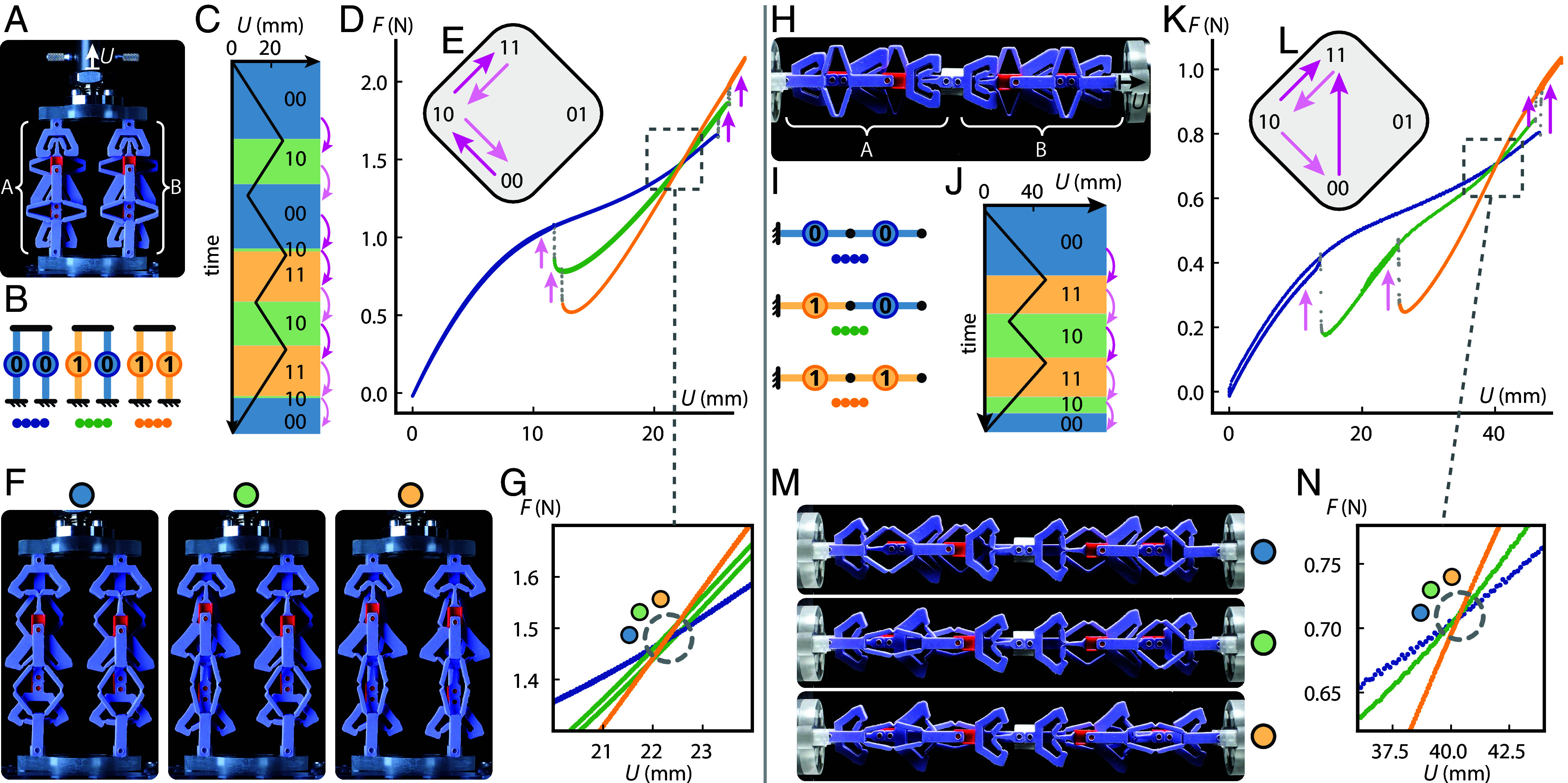
Experimentally observed collective behavior of countersnapping metamaterials. (*A*) Two parallel countersnapping elements controlled by displacement *U*. (*B*) Possible collective states of the system. (*C*) The binary state of the system (background color) as function of *U*, where bright (light) pink arrows indicate snapping events during loading (unloading). (*D* and *E*) Corresponding force–displacement curve (colors indicating state) and transition graph. (*F*) Snapshots of the {00}, {10} and {11} states at *U* = 22.3 mm near the intersection point of the force–displacement curves. (*G*) Close-up on the intersection point. (*H*) Two serially coupled countersnapping elements. (*I*) Possible collective states of the system. (*J*) State as function of *U*; note the avalanche transition {00}→{11}, where both countersnapping elements flip simultaneously. (*K* and *L*) Corresponding force–displacement curve and transition graph. (*M*) Snapshots of the {00}, {10} and {11} states at *U* = 40.3 mm near the intersection point. (*N*) Close-up on the intersection point.

We next show that mechanical metamaterials consisting of interacting countersnapping elements can switch between multiple internal configurations and stiffnesses. To explore the collective properties of such assemblies, we consider the two simplest configurations: parallel and serially coupled pairs of nearly identical countersnapping elements (Fig. 5). Each of the units can be in two configurations that we refer to as “0” (initial branch) and “1” (snapped branch), leading to collective configurations such as {00},{10},⋯ (Fig. 5 *B* and *I*) as observed in hysteron metamaterials (30, 32). To describe the transitions that can occur in our countersnapping metamaterials, we denote the critical extensions of countersnapping element *j* as $u_{j}^{+}$ for the 0→1 transition, and $u_{j}^{-}$ for the 1→0 transition.

We first consider the mechanical response of two countersnapping elements, A and B, placed in parallel, which effectively limits the interactions between both elements as their extension is directly controlled (Fig. 5*A*). Note that even though both elements show similar behavior, imperfections during manufacturing cause the value of $u_{\text{A}}^{+}$ to be about 0.7 mm lower than $u_{\text{B}}^{+}$, and $u_{\text{A}}^{-}$ is about 0.6 mm lower than $u_{\text{B}}^{-}$. Hence, when the extension is increased or decreased, the two units have a definite switching order. To demonstrate this, we design a specific driving protocol (Fig. 5*C*) that allows to access three collective states and all their respective transitions (Fig. 5 *D* and *E* and Movie S7). We note that the number of states that are accessible through our driving protocol with *N* countersnapping elements ranges from *N* + 1 to $2^{N}$, depending on the relative order of the critical extensions for the 0→1 and 1→0 transitions of the individual snapping elements (46). For example, a design with $u_{\text{A}}^{+}<u_{\text{B}}^{+}$ and $u_{\text{A}}^{-}>u_{\text{B}}^{-}$ would allow to access all four states (30, 47). For nearly identical countersnapping elements, the branches of the three configurations that are accessible, {00},{10}, and {11}, intersect at a single point in the force-extension plane (Fig. 5*D*, *F*, and *G*). The stiffness at this point only depends on the number of elements in state 0, and for assemblies of *N* = 2 and *N* = 3 elements (*SI Appendix*, Fig. S8), we show that all (N+1) possible stiffnesses are accessible. Hence, parallel assemblies of countersnapping elements allow flexibility in the ability to program the stiffness without influencing the observed external force and displacement.

**Fig. 6.**
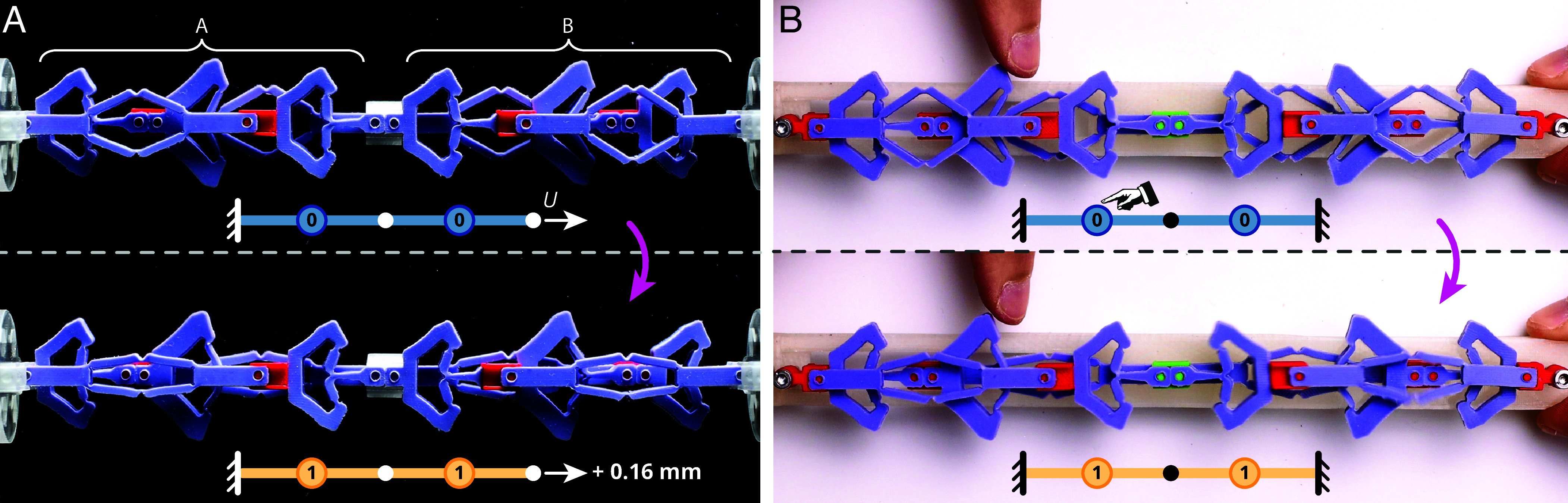
Avalanche transitions in serially coupled countersnapping elements. (*A*) Snapshots of two serially coupled countersnapping elements, steadily stretched under displacement control, before and after the sudden avalanche where both elements switch. (*B*) Snapshots of two countersnapping elements forming a chain whose total length is constant. Poking the *Left* element triggers a transition in both elements. Note that, despite the pair of countersnapping elements being oriented differently in panels (*A* and *B*), both arrangements correspond to the same (serial) coupling.

When two elements are coupled in series, they experience global interactions as the snapping of one unit influences all other units (16, 48). Different from ordinary snapping elements, serially coupled countersnapping elements experience an avalanche transition, directly switching from the {00} to the {11} during extension. Specifically, once one element switches phase 0→1, the concomitant increase in force triggers the instability of the other element in state 0 (Fig. 6*A* and Movie S8). By contrast, for serially coupled ordinary snapping structures, the first snapping event results in a force drop, which stabilizes the other elements in state 0 (16). We observed this simultaneous snapping also for three countersnapping elements placed in series (*SI Appendix*, Fig. S9) and believe that this phenomenon persists in longer chains as long as the level of imperfections is limited (48). When the load is sufficiently large, such an avalanche can also be triggered by manipulating one of the countersnapping elements so that it switches from state 0 to 1 (Fig. 6*B* and Movie S8). Upon unloading, we observe the elements to switch back to state 0 in steps, instead of collectively (Fig. 5 *K* and *L* and *SI Appendix*, Fig. S9 *D* and *E* and Movie S7). This asymmetric response extends the range of possibilities for nonreciprocal devices, e.g., unidirectional stick–slip actuators. We note that, similarly to parallel assemblies, *N* serially connected countersnapping elements allow for *N* + 1 collective stiffnesses (Fig. 5*K*, *M*, and *N* and *SI Appendix*, Fig. S9 and Movie S7).

These observations suggest that the self-intersection of the force–displacement curve is preserved in metastructures formed from countersnapping elements. For any assembly of countersnapping elements where one element is loaded to its intersection point, switching that element to the other state will affect the stiffness but not the external state of the assembly since the tension and elongation in that element is unchanged. These phenomena therefore highlight that countersnapping opens up a toolbox for mechanical metamaterials.

## Conclusion and Outlook

We demonstrated that mechanical countersnapping instabilities can be realized experimentally and that their unique self-intersecting force–displacement behavior allows new forms of mechanical intelligence, with potential applications in soft robots or computing metamaterials. Key to achieving countersnapping was a modular approach, where we translated the design of a highly nonlinear, self-intersecting force–displacement response to the design of a few weakly nonlinear building blocks which are then assembled to realize the targeted behavior. Combining multiple countersnapping units then leads to hierarchical designs with controllable snapping sequences and rapid collective snapping events, revealing another layer of complexity. As inversely designing matter with arbitrarily equilibrium curves has remained a challenging problem, we suggest that embracing this hierarchical paradigm opens up a route to achieving other kinds of complex—yet useful and targeted—responses.

Our work suggests that studying and leveraging the dynamic behaviors of snapping structures could lead to a more versatile level of programmability. Extending our observations of resonance avoidance through self-switching to more complex multistate assemblies could allow rapid switching between many more configurations and a targeted response to vibrations. Additionally, dynamical effects could be explored to promote or modify the rapid domain-wall propagation that occurs when countersnapping instabilities are coupled, leading to rich switching properties with potential applications in signal propagation and sensing (5, 49).

We close by listing key challenges. First, even though we were able to demonstrate countersnapping reliably, its implementation in larger assemblies and applications will likely require more compact designs with improved robustness. Second, further studies may enable countersnapping in other physical systems, such as fluidics and electronics, by engineering self-intersecting equilibrium curves associated with other pairs of conjugated variables such as pressure–volume, pressure–flow, or current–voltage. Third, the use of active materials could enable alternative ways to induce countersnapping, e.g., through heat (50), light (51), wetting (17) or magnetism (52), or enable reprogrammable responses that allow switching between counter- and regular snapping. We hope that our mechanical implementation of countersnapping is just the beginning and believe that, in time, countersnapping will achieve an impact comparable to that of conventional snapping instabilities.

## Materials and Methods

Each building block was fabricated by injecting a prepolymer mixture of silicone Smooth-Sil 945 (Smooth-on), into a mold assembled from three parts 3d-printed in VeroClear (Stratasys). The building blocks were assembled together using connectors 3d printed in polyactid acid. Assemblies of building blocks were modeled as networks of nonlinear extension springs, which were then solved using the arc-length numerical continuation scheme. Extension-driven tensile tests were conducted using uniaxial tensile test machines (Instron). The detailed description of the materials and methods is provided in *SI appendix*, section 1.

## Supplementary Material

Appendix 01 (PDF)

Movie S1. Tensile test of the countersnapping structure under displacement-controlled conditions. The displacement is increased then decreased, while the reaction force is measured. A sudden force jump is observed during the loading phase, and a self-intersecting force-displacement curve is obtained.

Movie S2. Tensile test of the countersnapping structure under force-controlled conditions. The tensile force is steadily increased by pouring water in a suspended cup, while the displacement is measured. A sudden contraction of the countersnapping structure (which lifts the cup) is observed while the cup is slowly filled.

Movie S3. Lifting a weight at rest on a platform and attached to the bottom of a countersnapping structure. The top of the countersnapping structure is steadily raised, while the weight elevation is measured. While raising the top of the structure, the weight, initially at rest, is suddenly lifted off the platform when the countersnapping instability is triggered.

Movie S4. Experimental comparison of the stick-slip behavior under cyclic loading of snapping and countersnapping structures. The regular snapping structure generates a back and forth actuation, while the countersnapping one generates an incremental unidirectional actuation that builds up with each cycle.

Movie S5. Experimental demonstration of programmable stiffness with unchanged equilibrium load and displacement. A countersnapping structure is preloaded to its intersection point by a suspended mass. The natural oscillation frequency of the system is measured. The frequency and the stiffness are shown to be programmable by switching between two states, which does not affect the force nor the elongation at equilibrium.

Movie S6. Experimental demonstration of self-switching stiffness upon resonance. A countersnapping structure is preloaded by a suspended mass to it intersection state then vertically vibrated from the top by a robotic arm, while the mass position is measured. When initially in the soft (stiff) state, the structure passively switches to the stiff (soft) state at the onset of resonance, which causes a reduction of the amplitude of the oscillations.

Movie S7. Collective behavior of countersnapping elements assembled in parallel and in series. By controlling the displacement to follow a certain signal over time, the state and the stiffness of the system can be programmed.

Movie S8. Avalanche transitions in serially-coupled countersnapping elements. Countersnapping elements connected in series snap together upon slowly stretching, manually poking or shaking.

## Data Availability

All simulation and experimental data are available on Zenodo, https://zenodo.org/records/15115687 (53). All other data are included in the article and/or supporting information.

## References

[1] K. Bertoldi, P. M. Reis, S. Willshaw, T. Mullin, Negative Poisson’s ratio behavior induced by an elastic instability. Adv. Mater. **22**, 361–366 (2010). 20217719 10.1002/adma.200901956

[2] S. Shan , Multistable architected materials for trapping elastic strain energy. Adv. Mater. **27**, 4296–4301 (2015). 26088462 10.1002/adma.201501708

[3] H. Yang, L. Ma, Multi-stable mechanical metamaterials by elastic buckling instability. J. Mater. Sci. **54**, 3509–3526 (2019).

[4] L. Wu, D. Pasini, In situ activation of snap-through instability in multi-response metamaterials through multistable topological transformation. Adv. Mater. **35**, 2301109 (2023). 10.1002/adma.202301109 37246407

[5] J. R. Raney , Stable propagation of mechanical signals in soft media using stored elastic energy. Proc. Natl. Acad. Sci. U.S.A. **113**, 9722–9727 (2016). 27519797 10.1073/pnas.1604838113 PMC5024640

[6] N. Nadkarni, A. F. Arrieta, C. Chong, D. M. Kochmann, C. Daraio, Unidirectional transition waves in bistable lattices. Phys. Rev. Lett. **116**, 244501 (2016). 27367390 10.1103/PhysRevLett.116.244501

[7] L. Jin , Guided transition waves in multistable mechanical metamaterials. Proc. Natl. Acad. Sci. U.S.A. **117**, 2319–2325 (2020). 31969454 10.1073/pnas.1913228117 PMC7007517

[8] B. Haghpanah, L. Salari-Sharif, P. Pourrajab, J. Hopkins, L. Valdevit, Multistable shape-reconfigurable architected materials. Adv. Mater. **28**, 7915–7920 (2016). 27384125 10.1002/adma.201601650

[9] Y. Liu , Multistable shape-reconfigurable metawire in 3D space. Extreme Mech. Lett. **50**, 101535 (2021).

[10] A. S. Meeussen, M. van Hecke, Multistable sheets with rewritable patterns for switchable shape-morphing. Nature **621**, 516–520 (2023). 37730868 10.1038/s41586-023-06353-5

[11] D. Melancon, A. E. Forte, L. M. Kamp, B. Gorissen, K. Bertoldi, Inflatable origami: Multimodal deformation via multistability. Adv. Funct. Mater. **32**, 2201891 (2022).

[12] C. M. Stafford , A buckling-based metrology for measuring the elastic moduli of polymeric thin films. Nat. Mater. **3**, 545–550 (2004). 15247909 10.1038/nmat1175

[13] J. H. Lee , Anisotropic, hierarchical surface patterns via surface wrinkling of nanopatterned polymer films. Nano Lett. **12**, 5995–5999 (2012). 23088734 10.1021/nl303512d

[14] T. Mei, C. Q. Chen, In-memory mechanical computing. Nat. Commun. **14**, 5204 (2023). 37626088 10.1038/s41467-023-40989-1 PMC10457397

[15] L. P. Hyatt, R. L. Harne, Programming metastable transition sequences in digital mechanical materials. Extreme Mech. Lett. **59**, 101975 (2023).

[16] J. Liu , Controlled pathways and sequential information processing in serially coupled mechanical hysterons. Proc. Natl. Acad. Sci. U.S.A. **121**, e2308414121 (2024). 38768343 10.1073/pnas.2308414121 PMC11145188

[17] H. Lee, C. Xia, N. X. Fang, First jump of microgel; Actuation speed enhancement by elastic instability. Soft Matter **6**, 4342–4345 (2010).

[18] D. Yang , Buckling of elastomeric beams enables actuation of soft machines. Adv. Mater. **27**, 6323–6327 (2015). 26389733 10.1002/adma.201503188

[19] T. Chen, O. R. Bilal, K. Shea, C. Daraio, Harnessing bistability for directional propulsion of soft, untethered robots. Proc. Natl. Acad. Sci. U.S.A. **115**, 5698–5702 (2018). 29765000 10.1073/pnas.1800386115 PMC5984517

[20] B. Gorissen, D. Melancon, N. Vasios, M. Torbati, K. Bertoldi, Inflatable soft jumper inspired by shell snapping. Sci. Robot. **5**, eabb1967 (2020). 33022625 10.1126/scirobotics.abb1967

[21] A. Nagarkar , Elastic-instability-enabled locomotion. Proc. Natl. Acad. Sci. U.S.A. **118**, e2013801118 (2021). 33602811 10.1073/pnas.2013801118 PMC7923676

[22] X. Cai, B. Tang, Mechanically controlled robotic gripper with bistability for fast and adaptive grasping. Bioinspir. Biomim. **18**, 014001 (2023). 10.1088/1748-3190/acaa7d 36575867

[23] J. T. B. Overvelde, T. Kloek, J. J. A. D’haen, K. Bertoldi, Amplifying the response of soft actuators by harnessing snap-through instabilities. Proc. Natl. Acad. Sci. U.S.A. **112**, 10863–10868 (2015). 26283372 10.1073/pnas.1504947112 PMC4568256

[24] Y. Tang , Leveraging elastic instabilities for amplified performance: Spine-inspired high-speed and high-force soft robots. Sci. Adv. **6**, eaaz6912 (2020). 32494714 10.1126/sciadv.aaz6912 PMC7209986

[25] L. C. Van Laake, A. Comoretto, J. T. Overvelde, On the coexistence of pressure regulation and oscillation modes in soft hysteretic valves. J. Fluids Struct. **126**, 104090 (2024).

[26] T. Schioler, S. Pellegrino, Space frames with multiple stable configurations. AIAA J. **45**, 1740–1747 (2007).

[27] D. P. Holmes, A. J. Crosby, Snapping surfaces. Adv. Mater. **19**, 3589–3593 (2007).

[28] G. Puglisi, L. Truskinovsky, Mechanics of a discrete chain with bi-stable elements. J. Mech. Phys. Solids **48**, 1–27 (2000).

[29] E. Ben-Haim, L. Salem, Y. Or, A. D. Gat, Single-input control of multiple fluid-driven elastic actuators via interaction between bistability and viscosity. Soft Rob. **7**, 259–265 (2020). 10.1089/soro.2019.0060 31891525

[30] M. Van Hecke, Profusion of transition pathways for interacting hysterons. Phys. Rev. E **104**, 054608 (2021). 34942848 10.1103/PhysRevE.104.054608

[31] H. Mofatteh , Programming multistable metamaterials to discover latent functionalities. Adv. Sci. **9**, 2202883 (2022). 10.1002/advs.202202883 PMC9685460 36253119

[32] J. Ding, M. van Hecke, Sequential snapping and pathways in a mechanical metamaterial. J. Chem. Phys. **156**, 204902 (2022). 35649852 10.1063/5.0087863

[33] L. C. van Laake, J. de Vries, S. Malek Kani, J. T. B. Overvelde, A fluidic relaxation oscillator for reprogrammable sequential actuation in soft robots. Matter **5**, 2898–2917 (2022).

[34] L. S. Novelino, Q. Ze, S. Wu, G. H. Paulino, R. Zhao, Untethered control of functional origami microrobots with distributed actuation. Proc. Natl. Acad. Sci. U.S.A. **117**, 24096–24101 (2020). 32929033 10.1073/pnas.2013292117 PMC7533839

[35] Z. G. Nicolaou, A. E. Motter, Mechanical metamaterials with negative compressibility transitions. Nat. Mater. **11**, 608–613 (2012). 22609557 10.1038/nmat3331

[36] J. Zha, Z. Zhang, Reversible negative compressibility metamaterials inspired by Braess’s paradox. Smart Mater. Struct. **33**, 075036 (2024).

[37] D. Braess, über ein Paradoxon aus der Verkehrsplanung. Unternehmensforschung **12**, 258–268 (1968).

[38] J. E. Cohen, P. Horowitz, Paradoxical behaviour of mechanical and electrical networks. Nature **352**, 699–701 (1991).

[39] C. M. Penchina, L. J. Penchina, The Braess paradox in mechanical, traffic, and other networks. Am. J. Phys. **71**, 479–482 (2003).

[40] T. E. Bruns, O. Sigmund, D. A. Tortorelli, Numerical methods for the topology optimization of structures that exhibit snap-through. Int. J. Numer. Methods Eng. **55**, 1215–1237 (2002).

[41] M. Wallin, N. Ivarsson, D. Tortorelli, Stiffness optimization of non-linear elastic structures. Comput. Methods Appl. Mech. Eng. **330**, 292–307 (2018).

[42] D. W. Pohl, Dynamic piezoelectric translation devices. Rev. Sci. Instrum. **58**, 54–57 (1987).

[43] S. Mohith, A. R. Upadhya, K. P. Navin, S. M. Kulkarni, M. Rao, Recent trends in piezoelectric actuators for precision motion and their applications: A review. Smart Mater. Struct. **30**, 013002 (2020).

[44] L. J. Kwakernaak, M. van Hecke, Counting and sequential information processing in mechanical metamaterials. Phys. Rev. Lett. **130**, 268204 (2023). 37450791 10.1103/PhysRevLett.130.268204

[45] J. Casals-Terre, A. Fargas-Marques, A. M. Shkel, Snap-action bistable micromechanisms actuated by nonlinear resonance. J. Microelectromech. Syst. **17**, 1082–1093 (2008).

[46] M. Mungan, M. M. Terzi, The structure of state transition graphs in systems with return point memory: I. General theory. Ann. Henri Poincaré **20**, 2819–2872 (2019).

[47] M. M. Terzi, M. Mungan, State transition graph of the Preisach model and the role of return-point memory. Phys. Rev. E **102**, 012122 (2020). 32795063 10.1103/PhysRevE.102.012122

[48] D. Shohat, M. van Hecke, Geometric control and memory in networks of bistable elements. arXiv [Preprint] (2024). https://arxiv.org/abs/2409.07804 (Accessed 30 September 2024).

[49] A. Pal, M. Sitti, Programmable mechanical devices through magnetically tunable bistable elements. Proc. Natl. Acad. Sci. U.S.A. **120**, e2212489120 (2023). 37011212 10.1073/pnas.2212489120 PMC10104571

[50] L. M. Korpas, R. Yin, H. Yasuda, J. R. Raney, Temperature-responsive multistable metamaterials. ACS Appl. Mater. Interfaces **13**, 31163–31170 (2021). 34164975 10.1021/acsami.1c07327

[51] M. R. Shankar , Contactless, photoinitiated snap-through in azobenzene-functionalized polymers. Proc. Natl. Acad. Sci. U.S.A. **110**, 18792–18797 (2013). 24190994 10.1073/pnas.1313195110 PMC3839691

[52] A. Abbasi, T. Chen, B. F. Aymon, P. M. Reis, Leveraging the snap buckling of bistable magnetic shells to design a refreshable braille dot. Adv. Mater. Technol. **9**, 2301344 (2024).

[53] P. Ducarme, B. Weber, M. van Hecke, J. T. B. Overvelde, Exotic mechanical properties enabled by countersnapping instabilities. Zenodo. 10.5281/zenodo.15115687. Deposited 3 April 2025. PMC12036990 40244676

