# Supplementary material for: Exotic mechanical properties enabled by countersnapping instabilities
Source: Proc Natl Acad Sci U S A. 2025 Apr 17;122(16):e2423301122. doi: 10.1073/pnas.2423301122 (PMC12036990; doi:10.1073/pnas.2423301122)
Supplement: Supplementary file 1 — Appendix 01 (PDF) [file pnas.2423301122.sapp.pdf]

Supporting Information for

Exotic mechanical properties enabled by countersnapping instabilities

Paul Ducarme, Bart Weber, Martin van Hecke*, Johannes T.B. Overvelde*

J.T.B. Overvelde
E-mail: b.overvelde@amolf.nl

This PDF file includes:

Supporting text
Figs. S1 to S9
Legends for Movies S1 to S8
SI References

Other supporting materials for this manuscript include the following:

Movies S1 to S8

Supporting Information Text

1. Materials and Methods

A. Manufacturing. The building blocks were manufactured by injecting a silicone elastomer prepolymer mixture (Smooth-Sil 945, Smooth-On) into a mold. Each mold is composed of three parts that were screwed tight together, thereby encasing a hollow cavity with the desired shape. Two metallic pins were inserted into the mold assembly to create the connecting features of the building block. Each mold was designed with one inlet and one outlet, allowing the prepolymers to be injected into the mold while letting air escape. After curing, the mold was disassembled carefully, which left the sample wedged in the central mold part. The sample was separated from the silicone cured in the inlet and outlet using a narrow blade, before using a stiff PLA block (with the same outline as the building block) to push and dislodge the sample out of the mold gently. More about the building block dimensions, mold design and fabrication process is shown in Fig. S1.

Mold fabrication and preparation The mold parts were 3d-printed using photocuring resin VeroClear for the model and SUP705 for support, using a PolyJet Eden260VS printer (Stratasys). After printing, the molds parts were placed in a KOH solution (5%) for 48 hours to dissolve the support material, then cleaned fully using a high-pressure waterjet. After drying each mold part using compressed air, the surfaces of the mold parts that are intended to be in contact with the curing silicone were treated by applying two layers of InhibitX (Smooth-On) using a soft brush. This is paramount to ensure a proper curing at the interface between the silicone and the mold surface. Once the InhibitX dried and before assembling the mold, the same surfaces were sprayed with mold release Ease Release 200 (Mann). When a mold was used again, it was cleaned using a high-pressure waterjet, then prepared following the same method: drying, InhibitX, mold release.

Material and cartridge preparation The prepolymer mixture that cures inside the mold was obtained as follows. First, the prepolymers part A and B were stirred for about 30 s separately. To every 100 g of part A 4 g of accelerant (Plat-Cat, Smooth-On) was added. The mix of part A and accelerant is mixed thourougly using a planetary mixer (Dispermill KK250) for 90 s, then poured into one compartment of a two-compartment cartridge (MIXPAC AF 400-01-10-01, Sulzer), while part B is poured into the other one. Before closing and sealing the compartements, the cartridge is placed vertically in a vacuum chamber for degassing (pressure of about -0.8 bar) for one hour.

Injection procedure To mix and inject the prepolymers into the mold, the cartridge was placed in a pneumatic dispenser gun (MIXPAC DP400-85, Sulzer) and a static mixer (QUADRO MFQ 05-24L, Sulzer) was mounted to its end and secured using a retaining nut (MIXPAC UM10-PP, Sulzer). To the Luer end of the static mixer was attached a female Luer (FTLL035), which was in turn connected to a silicone tube (5x8 mm) about 15 cm long, which was in turn connected to a male Luer (MLRL035) with a rotating ring lock (FSLLR) to which a dispensing conical needle (EFD7005009, 0.58 mm, Nordson) was screwed. Each assembled mold was secured vertically with the inlet hole facing downward, into which the conical needle was inserted. Each mold was slowly filled (about 1 min) until silicone started pouring from the outlet. Each mold was placed horizontally for about 12 hours at room temperature for curing.

Post-processing After removing each sample from their mold, all samples were postcured by leaving them at 80°C in a fan-assisted oven (UF30, Memmert). The samples were not stretched before the post-processsing was complete.

Assembly The building blocks were manually assembled together using 3d-printed PLA connectors, as shown in Fig. S3 and Fig. 1m.

B. Method to numerically predict the behavior of assemblies of nonlinear building blocks. To identify which building blocks should be assembled together to form a structure that countersnaps, we proceeded as follows.

- Each building block was characterized by measuring its force-displacement tensile curve (Fig. S2a-c).
- For each building block, a cubic Bezier curve was fitted to the measured force-displacement curve. A numerical nonlinear longitudinal spring was associated to each building block, with the tensile response of the spring defined by the cubic Bezier curve (Fig. S2a-c).
- The arc-length method was implemented to compute the equilibrium path of a structure consisting of five longitudinal nonlinear springs assembled in the network topology shown in Fig. S2d.
- For each combination of building blocks, the equilibrium path was computed and the direction of the snapping instability was determined (Fig. S2e).
- One numerical simulation predicting countersnapping was selected, then validated against the experimental tensile response (Fig. S2f-g).

Representation of each building block as a longitudinal nonlinear spring For the purpose of the numerical simulation, each building block is reduced to a longitudinal nonlinear spring, whose tensile behavior is represented by a cubic Bezier curve. A cubic Bezier curve is represented by the following parametric equations:

$$x_{[x_0,x_1,x_2,x_3]}(t) = x_0(1-t)^3 + x_1(1-t)^2 t + x_2(1-t)t^2 + x_3 t^3 \quad [1]$$

$$y_{[y_0,y_1,y_2,y_3]}(t) = y_0(1-t)^3 + y_1(1-t)^2 t + y_2(1-t)t^2 + y_3 t^3, \quad [2]$$

where t is parameter that runs from 0 to 1 to draw the Bezier curve, while x_0, x_1, x_2, x_3, y_0, y_1, y_2, y_3 are constant parameters that shape the curve. Upon choosing suitable values for x_0, x_1, x_2, x_3, y_0, y_1, y_2, y_3, the Bezier curve $(x(t), y(t))$ can be tuned to fit the experimental data (u_i, f_i). To ensure that the nonlinear spring yields no force when the extension is zero, x_0 and y_0 were set to 0. The 6 remaining fitting parameters $\boldsymbol{p} = [x_1, x_2, x_3, y_1, y_2, y_3]$ were found by minimizing the mismatch between the Bezier curve and the experimental curve. By enforcing $0 < x_1 < x_2 < x_3$, $x_{[0,x_1,x_2,x_3]}(t)$ is a monotonic function of t and therefore has an inverse, $t = x^{-1}_{[0,x_1,x_2,x_3]}(x)$. The Bezier curve can then be expressed as a function $\bar{y}_{\boldsymbol{p}}$ of x

$$y = y_{[0,y_1,y_2,y_3]}(x^{-1}_{[0,x_1,x_2,x_3]}(x)) = \bar{y}_{\boldsymbol{p}}(x), \quad [3]$$

and the minimization problem takes the following form

$$\min_{\boldsymbol{p}} \quad \sum_{i=1}^{n} \left(f_i - \bar{y}_{\boldsymbol{p}}(u_i)\right)^2, \quad [4]$$

$$\text{s.t.} \quad 0 < x_1 < x_2 < x_3, \quad [5]$$

where n is the number of experimental data points. For each building block, the experimental force-extension curve (u_i, f_i) considered is the one obtained during the second loading sequence. For the non-monotonic building blocks, this experimental curve was truncated so that (u_n, f_n) is the state at which the non-monotonic building block would be just after a snap-through instability. This helps obtain a better fit to the experimental data in the regime where the snapping building block will be used. For convenience, it is easier to express the force f_s in the spring s directly as a function of the extension u_s. To do this, we used $t = x^{-1}(x)$ (which is well-defined since we choose $0 < x_1 < x_2 < x_3$, forcing $u(t)$ to be a monotonic function of t), which leads to

$$f_s(u_s) = y(t) = y(x^{-1}(x)) = \bar{y}(x) = \bar{y}(u_s). \quad [6]$$

To compute the tangent stiffness k_s for spring s given its extension u_s, we used

$$k_s(u_s) = k(t) = k(x^{-1}(x)) = \bar{k}(x) = \bar{k}(u_s), \quad [7]$$

where $k(t) = y'(t)/x'(t)$ is the slope of the Bezier curve at the point $(x(t), y(t))$. The experimental force-extension curves along with their respective fitting Bezier curves are represented in Fig. S2a-c.

Simulating the nonlinear spring network We used the arc-length method (1, 2) to compute the equilibrium states of the assembled structure under tension. This method requires to compute the external nodal force vector f_i^{ext}, the internal nodal force vector f_i^{int} and the stiffness matrix K_{ij} of the spring structure depicted in Fig. S2d. The external nodal force vector $\boldsymbol{f}^{\text{ext}}$ is the set of external forces applied on each of the three degrees of freedom $\boldsymbol{q} = [x_{\text{A}}, x_{\text{B}}, x_{\text{C}}]$, and is therefore given by

$$\boldsymbol{f}^{\text{ext}} = \begin{pmatrix} 0 \\ 0 \\ F \end{pmatrix}, \quad [8]$$

where F is the tensile force applied on the bottom node of the structure, corresponding to the degree of freedom x_{C} (Fig. S2d) To compute the internal nodal force vector f_i^{int}, we start by expressing the elastic energy U of the structure as a function of the degrees of freedom $\boldsymbol{q} = [x_{\text{A}}, x_{\text{B}}, x_{\text{C}}]$

$$U(\boldsymbol{q}) = e_1(x_{\text{A}}) + e_3(x_{\text{B}} - x_{\text{A}}) + e_2(x_{\text{B}}) + e_1(x_{\text{C}} - x_{\text{B}}) + e_2(x_{\text{C}} - x_{\text{A}}), \quad [9]$$

where $e_i(.)$ is the elastic energy stored in a nonlinear spring of type i. The internal nodal force vector f_i^{int} is then given by

$$f_i^{\text{int}}(\boldsymbol{q}) = \frac{\partial U}{\partial q_i}, \quad [10]$$

or in a more developed form

$$f_1^{\text{int}} = f_1(x_{\text{A}}) - f_3(x_{\text{B}} - x_{\text{A}}) - f_2(x_{\text{C}} - x_{\text{A}}) \quad [11]$$

$$f_2^{\text{int}} = f_2(x_{\text{B}}) + f_3(x_{\text{B}} - x_{\text{A}}) - f_1(x_{\text{C}} - x_{\text{B}}) \quad [12]$$

$$f_3^{\text{int}} = f_2(x_{\text{C}} - x_{\text{A}}) + f_1(x_{\text{C}} - x_{\text{B}}), \quad [13]$$

where $f_i(.) := e_i'(.)$ is the derivative of the elastic energy $e_i(.)$. The stiffness matrix $K_{ij}(\boldsymbol{q})$ is given by

$$K_{ij}(\boldsymbol{q}) = \frac{\partial^2 U}{\partial q_i \partial q_j} = \frac{\partial f_i^{\text{int}}(\boldsymbol{q})}{\partial q_j}, \quad [14]$$

or in a more developed form

$$K_{11} = k_1(x_\mathrm{A}) + k_3(x_\mathrm{B} - x_\mathrm{A}) + k_2(x_\mathrm{C} - x_\mathrm{A}) \quad [15]$$

$$K_{12} = -k_3(x_\mathrm{B} - x_\mathrm{A}) = K_{21} \quad [16]$$

$$K_{13} = -k_2(x_\mathrm{C} - x_\mathrm{A}) = K_{31} \quad [17]$$

$$K_{22} = k_1(x_\mathrm{C} - x_\mathrm{B}) + k_3(x_\mathrm{B} - x_\mathrm{A}) + k_2(x_\mathrm{B}) \quad [18]$$

$$K_{23} = -k_1(x_\mathrm{C} - x_\mathrm{B}) = K_{32} \quad [19]$$

$$K_{33} = k_1(x_\mathrm{C} - x_\mathrm{B}) + k_2(x_\mathrm{C} - x_\mathrm{A}), \quad [20]$$

where $k_i(.) := f_i'(.) = e_i''(.)$ is the derivative of the tensile force $f_i(.)$. Given these three ingredients, the arc-length method allowed us to retrieve the entire equilibrium path connected to the initial configuration. This path is composed of equilibrium points $(\boldsymbol{q}_\mathrm{eq}, F)$ whose stability can be assessed by evaluating the stiffness matrix of the entire structure, $\boldsymbol{K}(\boldsymbol{q}_\mathrm{eq})$. The stability of an equilibrium point depends on the loading mode. If one drives the force, an equilibrium point is stable if and only if all the eigenvalues of the stiffness matrix $\boldsymbol{K}(\boldsymbol{q}_\mathrm{eq})$ are positive. If one drives the extension, an equilibrium point is stable if and only if the eigenvalues of the reduced stiffness matrix $\bar{\boldsymbol{K}}(\boldsymbol{q}_\mathrm{eq})$ are positive. The reduced stiffness matrix is defined as the stiffness matrix $\boldsymbol{K}$ from which the row and column corresponding to the degree of freedom controlled have been removed. Note that when an equilibrium point is stable under force control, it will necessary be stable under extension control, but the opposite is generally not true.

Computational details We scanned all the combinations of nonlinear springs and simulated every single assembly. In total, we performed 324 simulations (9 softening building blocks $\times$ 4 stiffening building blocks $\times$ 9 snapping building blocks). The simulations were run on a desktop PC with the following specifications: Windows 10 Enterprise, Intel(R) Core(TM) i7-6700 CPU @ 3.40GHz, 16GB RAM. The total time needed to simulate all the assemblies was 7 min 26 s. The average time per assembly was 1.38 s (standard deviation of 0.39 s). The median time was 1.27 s. The arclength method involves evaluating the residual of the system, evaluating the jacobian of the system and solving linear systems iteratively. Together, these three tasks accounted for about 75% of the computation time. Cumulatively, over all assembly simulations, system residuals and jacobians were evaluated 338582 times each and 561291 linear systems (3×3) were solved.

Overview of the scanning results, identification of promising combinations and experimental validation For computed each equilibrium path, we extracted the force-displacement curve which allowed us to determine the direction of the first snapping instability ΔF (if any). The direction of the snapping instability is defined as the sudden change in reaction force F caused by a snapping instability under displacement-driven conditions. If negative, the instability causes the reaction force to drop (regular snapping); if positive, the instability causes the reaction force to jump (countersnapping). For many combinations, the structure formed is not subject to any instability under displacement-driven conditions (the force-displacement curve does not 'curve back'), or even under force-driven conditions (the force-displacement curve is monotonic). In these two latter cases, we cannot define any sudden force change.

The results of the simulations are shown in Fig. S2e. As show in Fig. S2e, a few building block assemblies are predicted to show a countersnapping instability (purple-colored squares). We selected a combination with a relatively pronounced predicted force jump which corresponds to a specific set of design parameters (as highlighted in magenta in Fig. S2e-f). Once we manufactured extra softening and stiffening building blocks, the entire structure was built, tested physically, and compared to the numerical prediction. As shown in Fig. S2g, the spring model correctly predicts the countersnapping instability. The slight discrepancy is mainly caused by the small difference in behaviors between samples with the same design, and the fact that the building blocks are not aligned along exactly one line as it is the case in the simulation.

C. Testing methods. All extension-driven tensile tests were performed using a vertical uniaxial testing machine (model 5965, Instron) equipped with a 100-N load cell, except for the the tensile tests involving countersnapping elements combined in series that were performed using a horizontal uniaxial testing machine (custom model, controller type 5900, Instron) equipped with a 10-N load cell.

Tensile test of the individual building blocks (displacement-controlled), Fig. 1j-l Each sample was attached to two PLA connectors (similar to the one shown in Fig. S3a), which were then clamped by the testing machine clamps. Each sample was subjected to a cyclic loading (30 s starting hold at 0 mm, 4 loading-unloading cycles at 1 mm/s). Acquisition rate: 10 Hz.

Tensile test of the countersnapping structure (displacement-controlled), Fig. 2a-c The countersnapping structure was attached to the testing machine by clamping its PLA end connectors (similar to the one shown in Fig. S3a). Each sample was subjected to a cyclic loading (30 s starting hold at 0 mm, 4 loading-unloading cycles at 1 mm/s). Acquisition rate: 10 Hz.

Force-driven tensile test of the countersnapping structure, Fig. 2d-f The countersnapping structure was inserted into a custom-made sliding mechanism allowing it to deform only uniaxially (preventing bending and swinging motion). The mechanism consists of a slider able to move up and down in the slots of two parallel acrylic plates. The slider featured a similar connecting design to the one shown in Fig. S3a. We attached the bottom of the countersnapping structure to the slider. The top of the countersnapping structure was attached to the fixed connector, located in between both plates. The sliding mechanism was attached vertically from the top of the 100-N load cell. We attached a plastic cup to the slider using an thin inextensible rope.

The force was measured over time using the load cell (acquisition rate = 100 Hz). The extension was monitored by tracking an aruco marker, sticked to the slider, and recording its position using a high-resolution camera (EOS 850D, Canon) at 50 frames per second. Tracking was performed using the blob detection algorithm of the OpenCV Python library. Water was manually poured into the cup using a syringe. Note that we assumed that the applied force value (weight of the cup and water) was the same as the force measured using the load cell, except around the countersnapping instability which caused the system to enter a temporary dynamic regime for about 0.5 s. The force values measured by the load cell during that short dynamic period were discarded and replaced by values deduced from a linear regression (time-force) based on the force evolution data collected a few second before and after the dynamic phase.

Lift-weight experiment, Fig. 2g-i The weight was a custom-made 3d-printed PLA block to which flat cylindrical calibrated weights can be added. The total weight was about 0.83 N. The weight was placed on a horizontal platform right underneath the top clamp of the vertical tensile machine. The platform was covered by a sheet of paper to mitigate the effect of electrostatic forces between the platform and the PLA weight. The weight was attached to the bottom of the countersnapping structure using a thin, inextensible rope. The top of the countersnapping structure was clamped via the top end connector to the movable head of the uniaxial tensile machine. The top clamp was raised by 26mm at 0.05 mm/s, then lowered back to its initial level at 2.00 mm/s. This cycle was repeated 4 times. The top displacement was measured using the uniaxial vertical tensile machine (acquisition rate: 100 Hz), while the weight elevation was measured by tracking two black dots marked on the weight using a using a high-resolution camera (EOS 850D, Canon) at 50 frames per second.

Stick-slip experiments, Fig. 3 The mechanical structures were cyclically loaded using the linear motion of a programmed robotic arm (UR5e, Universal Robots), pulling on the end connector via a soft elastic rubber band (measured stiffness: 0.003 N/mm). The loaded end connector was 3d-printed in VeroClear (Stratasys). The foam block was cut from a white foam board. It is 51 mm long, 34 mm wide, 5 mm thick and weighed 0.7 g. One side was made rougher by scraping it against sand paper. The other side was kept smooth and intact, and was in contact with the bottom surface (anodized aluminum sheet). The desired amount of roughness (and friction) was tuned by incrementally scraping against sand paper up to the point where stick behavior was observed during slow motion (regular loading) and slip behavior during fast motion (snapping). The same foam block with the same level of roughness was used for all experiments. The regular snapping structure was a non-monotonic building block with parameters $d = 9$ mm, $\theta = 60^{o}$. The change in drift, defined as the change of distance between the foam block and the contact point (end connector/foam block) was computed by tracking both the foam block position and contact point position using high-resolution cameras (EOS 850D, Canon), recording simultaneously at 50 fps. The foam block position was monitored by tracking black markers marked on the top of the foam block (top view camera), while the contact point position was computed by tracking three holes in the end connector (side view camera).

Free oscillation test, Fig. 4c-e The set-up was the same as for the force-driven tensile test. The amount of water added to the cup was tuned so that the extension of the countersnapping structure is the same for both stable configurations. This was ensured by comparing photographs of both configurations taken from the same camera set-up. The change in extension ΔU compared to the preloaded configuration was monitored by tracking the aruco marker and recording its position using a high-resolution camera (EOS 850D, Canon) at 50 frames per second.

Forced oscillation test, Fig. 4f-h The set-up was the same as for the force-driven tensile test, except for two aspects. First, the weight consists of dumbbell composed of a PLA slider to which is attached a threaded rod carrying washers and nuts to tune the load to the desired level. Second, the system was attached vertically to the end effector of a 6-axis robotic arm (UR5e, Universal Robots). The oscillating displacement input and output were monitored by sticking circular markers to the top and bottom of the structure, and recording them using a high-resolution camera (EOS 850D, Canon) at 50 frames per second. The input and output displacements were measured by detecting the vertical position of the markers on every frame using the blob-detection algorithms of the OpenCv library.

Parallel-coupled countersnapping elements tensile test, Fig. 5a-g, Fig. S8 countersnapping elements were combined using custom-made connectors, 3d-printed in VeroClear (Stratasys), that were attached to the uniaxial testing machine. Force and displacement values were measured at 100 Hz. Loading speed was 1 mm/s.

Serially-coupled countersnapping element tensile tests, Fig. 5h-n, Fig. 6a, Fig. S9 To combine countersnapping elements in series, connectors were 3d-printed in VeroClear using a PolyJet Eden260VS printer (Stratasys). Those connectors were designed in such a way that they connected the stiffening (and softening) building blocks of adjacent countersnapping elements at the same level. Those connectors were designed with long feet that allowed them to slide on the (horizontal) surface (anodized aluminum sheet) without letting the countersnapping elements themselves touch the surface. To mitigate the effect of friction when three countersnapping elements were connected in series, the connectors were sliding on microscope slices brushed with a thin layer of oil (Morlina S2 BL, Shell). The force and displacement were measured at 100 Hz. For the tests with two countersnapping elements, the loading speed was 2 mm/s. For the tests with three countersnapping elements, the loading speed was 3 mm/s.

Serially-coupled countersnapping element constant-length tests, Fig. 6b The two countersnapping elements were connected in series using a 3d-printed PLA connector in such a way that the stiffening (and softening) building blocks of each countersnapping elements were connected at the same level. The chain that they formed was prestretched in a custom-made, 3d-printed PLA linear stage that allowed for tuning the desired total length, then keeping it fixed for the duration of the test. The prestretch

applied was about 47 mm. The behavior of the structure was recorded using a high-resolution camera (EOS 850D, Canon) at 25 fps.

2. Additional conditions to obtain countersnapping

The force-displacement curves of the individual nonlinear springs must satisfy some conditions in order to obtain a countersnapping instability. To derive these conditions, we first determine the equilibrium points of the system, then study the stability of these equilibria. To determine to equilibrium points of the system, we first express the total potential energy $\Pi(.,.,.)$ of the system described in Fig. S2d as function of the three degrees of freedom (DOF) $x_\mathrm{A}, x_\mathrm{B}, x_\mathrm{C}$:

$$\Pi(x_\mathrm{A}, x_\mathrm{B}, x_\mathrm{C}) = e_1(x_\mathrm{A}) + e_2(x_\mathrm{B}) + e_3(x_\mathrm{B} - x_\mathrm{A}) + e_1(x_\mathrm{C} - x_\mathrm{B}) + e_2(x_\mathrm{C} - x_\mathrm{A}) - x_\mathrm{C} F, \quad [21]$$

where $e_i(.)$ is the elastic energy stored in a nonlinear spring of type i, F is the force applied on the bottom node. Equilibrium points are stationary points of the total potential energy Π, so they must satisfy the following conditions

$$0 = \frac{\partial \Pi}{\partial x_\mathrm{A}} = f_1(x_\mathrm{A}) - f_3(x_\mathrm{B} - x_\mathrm{A}) - f_2(x_\mathrm{C} - x_\mathrm{A}) \quad [22]$$

$$0 = \frac{\partial \Pi}{\partial x_\mathrm{B}} = f_2(x_\mathrm{B}) + f_3(x_\mathrm{B} - x_\mathrm{A}) - f_1(x_\mathrm{C} - x_\mathrm{B}) \quad [23]$$

$$0 = \frac{\partial \Pi}{\partial x_\mathrm{C}} = f_1(x_\mathrm{C} - x_\mathrm{B}) + f_2(x_\mathrm{C} - x_\mathrm{A}) - F, \quad [24]$$

where the tension force $f_i(.) := e_i'(.)$ is the derivative of the elastic energy e_i. From Eqs (22, 23), we have

$$f_1(x_\mathrm{A}) - f_1(x_\mathrm{C} - x_\mathrm{B}) = f_2(x_\mathrm{C} - x_\mathrm{A}) - f_2(x_\mathrm{B}) \quad [25]$$

In the following, we show that by assuming that $f_1(.)$ and $f_2(.)$ are monotonically increasing functions, $x_\mathrm{A} = x_\mathrm{C} - x_\mathrm{B}$ is necessarily true, which is a useful result that will simplify the stability analysis we will conduct next. For the sake of the proof, let's assume $x_\mathrm{A} > x_\mathrm{C} - x_\mathrm{B}$. In that case, given the monotonicity of $f_1(.)$ and $f_2(.)$, $x_\mathrm{C} - x_\mathrm{A} > x_\mathrm{B}$ in order to maintain Eq. (25) balanced, which contradicts our initial assumption. Therefore, $x_\mathrm{A} = x_\mathrm{C} - x_\mathrm{B}$. This result means that our system has effectively only two DOF. We can choose u_1 and u_2 (stretch of springs s_1 and s_2) to represent those. The total potential energy $\Omega(.,.)$ of the system can be then expressed as follows,

$$\Omega(u_1, u_2) = 2e_1(u_1) + 2e_2(u_2) + e_3(u_2 - u_1) - (u_1 + u_2)F, \quad [26]$$

and the equilibrium conditions take the following form

$$0 = \frac{\partial \Omega}{\partial u_1} = 2f_1(u_1) - f_3(u_2 - u_1) - F \quad [27]$$

$$0 = \frac{\partial \Omega}{\partial u_2} = 2f_2(u_2) + f_3(u_2 - u_1) - F. \quad [28]$$

When the above equations are rewritten as follows

$$\left(f_1(u_1) + f_2(u_2)\right)/2 = F/2 \quad [29]$$

$$f_1(u_1) - f_2(u_2) = f_3(u_2 - u_1), \quad [30]$$

it is clear that half the global force F is the average of f_1 and f_2, while f_3 is the difference between f_1 and f_2, thereby justifying the geometric construction employing rectangles introduced in the main text to represent equilibrium configurations of the system.

To study the stability of the equilibrium points, we construct the hessian matrix $\boldsymbol{K}$ of the system, that is the matrix of the second derivatives of Ω with respect to the two degrees of freedom $\boldsymbol{q} = [u_1, u_2]$,

$$\boldsymbol{K} = \frac{\partial^2 \Omega}{\partial \boldsymbol{q}^2} = \begin{pmatrix} 2k_1(u_1) + k_3(u_3) & -k_3(u_3) \\ -k_3(u_3) & 2k_2(u_2) + k_3(u_3) \end{pmatrix}, \quad [31]$$

where the stiffness $k_i(.) := f_i'(.) = e_i''(.)$ is the derivative of the tension force f_i, and $u_3 := u_2 - u_1$. By applying Sylvester's criterion, we derive that the system is unstable if and only if

$$k_3(u_3) < -2\frac{k_1(u_1)k_2(u_1)}{k_1(u_1) + k_2(u_2)}, \quad [32]$$

where the right-hand side is always negative given the monotonicity of f_1 and f_2.

This means that two additional conditions must be met when drawing the force-displacement curves during the third step of the geometric construction detailed in the main text (Fig. 1i):

- for the system to be on the verge of becoming unstable, the slope of $f_3(u_3)$ must equal the (negative) right-hand side of Ineq. (32),

- for the system to be stable after the sudden reconfiguration, the slope of $f_3(u_3)$ must be greater than the (negative) right-hand side of Ineq. (32).

It is important to note that the slope of $f_3(u_3)$ must approach the prescribed negative critical value by decreasing as the system is loaded. This implies that the stiffness k_1 (slope of f_1) must be greater than the stiffness k_2 (slope of f_2) when reaching the critical equilibrium point. Otherwise, the spring s_3 will see its elongation decrease when approaching the critical point, thereby making the desired critical point actually unreachable by simply loading the system from the starting configuration.

3. Geometric construction of local force-displacement curves leading to countersnapping during unloading

The geometric construction of local force-displacement curves introduced in the main text can also be used to identify individual force-displacement curves leading to countersnapping during unloading, instead of loading.

We consider the same nonlinear spring network as illustrated in Fig. S4a. As shown in Fig. S4b, we first draw a rectangle whose top-left and bottom-right corners correspond to the states of springs s_1 and s_2, just before the instability. Next, because we want the restabilized state to be more elongated (and at the same level of critical force), we draw a second rectangle whose center is shifted, horizontally to the *right* compared to the first rectangle. Again, the top-left and bottom-right corners correspond to the state of s_1 and s_2, just after the instability. Since the widths and heights of both rectangles correspond to the states of the spring s_3 before and after the instability, two additional markers can be positioned in the force-displacement plane (green dot and green star). The three individual force-displacement curves are then plotted through their corresponding markers. The same two conditions as detailed in the previous section must be met by the curve $f_3(u_3)$ to ensure that the first rectangle describes a critical equilibrium point. Importantly, the slope of $f_3(u_3)$ must approach the prescribed negative critical value by decreasing as the system is unloaded. This implies that the stiffness k_1 (slope of f_1) must be *smaller* than the stiffness k_2 (slope of f_2) when reaching the critical equilibrium point. Otherwise, the spring s_3 will see its elongation decrease when approaching the critical point, thereby making the desired critical point actually unreachable by simply unloading the system from the stretched configuration.

To confirm that such set of curves leads to countersnapping, we numerically simulate the nonlinear spring network (using the arc-length method, detailed in the Materials and Method section 1.B) using the exact curves drawn in Fig. S4b, and compute the force-displacement curve of the system. As shown in Fig. S4c, if we start unloading the system from the preloaded configuration (marked by the triangle) by reducing the tensile force, the elongation slowly decreases. Upon reaching a critical force level (marked by the circle), the system becomes unstable and reconfigures suddenly in a more elongated state (marked by a star). Less importantly, we can notice that the system will later undergo a second snap-through instability (this time leading to a typical decrease in elongation) if the force is reduced further. This particular system shares similar properties as the countersnapping structure used in the main text: self-intersecting force-displacement curve, force drop if one unloads under displacement-driven conditions, etc).

The individual curves used for the simulation are piecewise linear curves whose corners have been rounded off using a quadratic function. They can be expressed mathematically as follows,

$$f(u) = \sum_{i=0}^{n-1} L_i(u)(k_i u + p_i) + Q_i(u)(a_i u^2 + b_i u + c_i), \qquad [33]$$

where f is the force, u is the displacement, k_i is the slope of the ith segment and $n \geq 2$ is the number of segments composing

the (not-smoothened) piecewise linear curve. The quantities $L_i(u)$, $Q_i(u)$, p_i, a_i, b_i and c_i are defined as follows,

$$L_i(u) = \begin{cases} \begin{cases} 1 & u < u_i - \epsilon \\ 0 & \text{else} \end{cases} & i = 0 \\ \begin{cases} 1 & u_{i-1} + \epsilon \leq u < u_i - \epsilon \\ 0 & \text{else} \end{cases} & 1 \leq i \leq n-2 \\ \begin{cases} 1 & u_{i-1} + \epsilon \leq u \\ 0 & \text{else} \end{cases} & i = n-1 \end{cases} \quad [34]$$

$$Q_i(u) = \begin{cases} \begin{cases} 1 & u_i - \epsilon \leq u < u_i + \epsilon \\ 0 & \text{else} \end{cases} & 0 \leq i \leq n-2 \\ 0 & i = n-1 \end{cases} \quad [35]$$

$$p_i = \begin{cases} 0 & i = 0 \\ p_{i-1} + u_{i-1}\left(k_{i-1} - k_i\right) & 1 \leq i \leq n-1 \end{cases} \quad [36]$$

$$a_i = \begin{cases} \frac{k_{i+1} - k_i}{4\epsilon} & 0 \leq i \leq n-2 \\ 0 & i = n-1 \end{cases} \quad [37]$$

$$b_i = \begin{cases} \frac{k_i(u_i+\epsilon) - k_{i+1}(u_i - \epsilon)}{2\epsilon} & 0 \leq i \leq n-2 \\ 0 & i = n-1 \end{cases} \quad [38]$$

$$c_i = \begin{cases} \frac{(k_{i+1} - k_i)(u_i - \epsilon)^2}{4\epsilon} + p_i & 0 \leq i \leq n-2 \\ 0 & i = n-1 \end{cases}, \quad [39]$$

where u_i is the displacement at which the (not-smoothened) piecewise linear curve goes from slope k_i to k_{i+1} and ϵ is a parameter to tune the smoothing which must satisfy the following constraint,

$$\epsilon < \min \left\{ \min_{1 \leq i \leq n-2} \left\{ \frac{u_i - u_{i-1}}{2} \right\}, u_0 \right\} \quad [40]$$

The force-displacement curves of springs s_1, s_2 and s_3 are defined using the following parameters,

- spring s_1: $\boldsymbol{k} = [1.9438; 0.01723; 3.0603]$, $\boldsymbol{u} = [0.6542; 3.9302]$, $\epsilon = 0.2$
- spring s_2: $\boldsymbol{k} = [0.18401; 3.0934]$, $\boldsymbol{u} = [3.0766]$, $\epsilon = 0.2$
- spring s_3: $\boldsymbol{k} = [4.7619; -0.6042; 0.61]$, $\boldsymbol{u} = [0.2315; 1.9711]$, $\epsilon = 0.2$.

4. Sensitivity analysis of the geometric parameters

In this section, we conduct a two-step analysis to investigate the interplay between geometric parameters and the countersnapping effect.

For the first step, we investigated how the force-displacement curves of the individual building blocks are influenced by the geometric parameters (Fig. S5a-e), to map the geometric parameters of each building block to the shape of their force-displacement curve. This mapping was established by analyzing how the control points' coordinates of the Bezier curves that fit the experimental tensile test data of the individual building blocks are modified as one geometric parameter is varied. For any value of a geometric parameter, we estimate the force-displacement curve of the concerned building block by constructing a cubic Bezier curve whose control points' coordinates are determined by the interpolation or extrapolation of the discrete set of measured samples.

For example, for the parameter a (that is, the length of the inclined beam of the softening unit), we identified the three softening building blocks for which a was varied ($a = [4, 5, 6]$ mm) while keeping the other parameter constant, namely $b = 1$ mm. For each of the three softening building blocks (respectively characterized by $(a, b) = [(4, 1), (5, 1), (6, 1)]$ mm), we determined the control points' coordinates of the cubic Bezier curve that fits their force-displacement tensile curve. To estimate the force-displacement curve for a new value of a, let us say $a = 4.5$ mm, we constructed a new cubic Bezier curve for which each of its control points' coordinates is estimated by interpolating between the corresponding known coordinates when $a = 4$ mm and $a = 5$ mm. The estimated force-displacement curves obtained for values of a ranging from 3 to 7 mm, with $b = 1$ mm are shown in Fig. S5a. The same process is repeated for parameters b, c, d and θ, for which results are shown in Fig. S5b, c, d and e, respectively. Note that for the softening and non-monotonic building blocks, the value of the parameter that is kept constant (while the other one is varied) is the value used for the current countersnapping design structure with $a = 4$ mm, $b = 1$ mm, $d = 9$ mm and $\theta = 60^{o}$.

For the second step, we use the mapping established in the first step to investigate the magnitude of the countersnapping effect under displacement-driven conditions as a function of the geometric parameters (Fig. S5f-j). We define this magnitude as the sudden change in force ΔF due to the instability. If the force jumps due a countersnapping instability, the magnitude is positive ($\Delta F > 0$). If the force drops due a regular instability, the magnitude is negative ($\Delta F < 0$). If there is no instability, we cannot define a magnitude (ΔF is undefined). We studied each parameter by independently varying each parameter with respect to the reference spring network (Fig. 1e), and show the magnitude ΔF as a function of each parameter in Fig. S5f-j. Note that the bar under the x-axis indicates the range for which data fall within tested parameters, where results outside this range represent less accurate extrapolated results.

From the results in Fig. S5 we can extract how sensitive the countersnapping effect is with respect to the geometric parameters. Importantly, changing the value of a parameter can continuously decrease the sudden change in force ΔF from a positive (countersnapping, purple regions) to a negative (regular snapping, green regions) value. Interestingly, tuning a geometric parameter can also remove any instability from the system (no instability, gray regions).

For example, the thickness of the central flexure of the softening unit, b, has a strong impact on the countersnapping effect. If b is slightly decreased, the system is not able to undergo an instability. If b is slightly increased, the force jumps quickly becomes a force drop (Fig. S5g). This influence makes sense intuitively. If b is large, the softening unit becomes a very stiff element (Fig. S5b), thereby leading the system to destabilize and reconfigure at a lower global displacement, at which the stiffening units are not yet in their stiffening regime. Inversely, if b is small, the softening unit becomes a very soft element (Fig. S5b), thereby requiring a larger global displacement to approach the instability, which eventually becomes unreachable as soon as the stiffening units stiffen. By constrast, the length of the beams of the non-monotonic unit, d, has a significantly lower influence on the countersnapping effect. The parameter d mostly affects the 'valleys' of the non-monotonic curves, whereas it almost does not alter the 'peaks' (Fig. S5d), which are crucial as they govern the onset of instability.

5. Countersnapping upon both loading and unloading

In this section we discuss in detail the constraints on potential force displacement curves that realize countersnapping upon both loading and unloading (Fig. S6).

A naive approach to construct countersnapping upon loading and unloading is based on two stable branches, with the secondary branch shifted upwards and to the left compared to the primary one in the force-displacement plane. Assuming both stable branches are connected by an unstable branch, one might be tempted to draw an unstable path like in Fig. S6a,b. However, we can immediately exclude the existence of such force-displacement curves based on the existing 'folding rules' (3). This theory tells us that each time the curve folds clockwise (by reaching a force extremum, or a displacement extremum), the number of unstable modes (under force-, or displacement-driven conditions) is increased by one. Inversely, each time the curve folds counter-clockwise, the number of unstable modes under the respective loading driving conditions is decreased by one. Since the primary branch is stable, the curve must fold clockwise and counter-clockwise the same number of times before reaching the second stable branch, thereby making the curve drawn in Fig. S6a not realizable by a physical system. Also, the first fold must necessarily be clockwise since the number of unstable modes cannot be lower than zero, which also excludes the curve drawn in Fig. S6b.

The two stable branches could still potentially be connected by drawing a curve that folds first clockwise then counter-clockwise (Fig. S6c). However, while respecting these 'folding rules' is necessary, it is not sufficient to guarantee that such a curve represents a physical elastic system: snapping instabilities at constant displacement should also cause a transition from a high elastic energy state to a lower elastic energy state. For the curve depicted in Fig. S6c, satisfying these conditions for both the loading and unloading snapping transitions is impossible, as we will demonstrate next.

First, we calculate the elastic energy $E_{\text{elastic}}(X)$ stored in a state X along the equilibrium curve using

$$E_{\text{elastic}}(X) = \int_0^X F(t)\frac{dU(t)}{dt}dt, \qquad [41]$$

where t is the curve parameter, which monotonically increases as one moves along the parametric curve $(U(t), F(t))$. Eq. (41) defines an integral quantity that can be decomposed as a combination of areas E_i (Fig. S6d). For the point X_c^+, the critical point reached during loading, the elastic energy is

$$E_{\text{elastic}}(X_c^+) = E_1 + E_2 + E_3 + E_4 + E_5.$$

For the point X_r^+, the restabilization point reached just after the loading snapping transition, the elastic energy is

$$E_{\text{elastic}}(X_r^+) = E_{\text{elastic}}(X_c^+) - E_6 + E_9 + E_{10} + E_4.$$

Therefore, for the snapping transition upon loading to release elastic energy, we must have

$$0 < E_{\text{elastic}}(X_c^+) - E_{\text{elastic}}(X_r^+) \Leftrightarrow E_6 > E_4 + E_9 + E_{10}. \qquad [42]$$

Similarly, for the point X_c^-, the critical point reached during unloading, the elastic energy is

$$E_{\text{elastic}}(X_c^-) = E_1 + E_4 - E_8 - E_6 - E_7$$

For the point X_r^-, the restabilization point reached just after the unloading snapping transition, the elastic energy is

$$E_{\text{elastic}}(X_r^-) = E_1.$$

Therefore, for the snapping transition upon unloading to release elastic energy, we must have

$$0 < E_{\text{elastic}}(X_c^-) - E_{\text{elastic}}(X_r^-) \Leftrightarrow E_4 > E_6 + E_7 + E_8. \quad [43]$$

Since $E_i > 0$, condition (43) is incompatible with the energy release condition for the snapping transition upon loading (42). This contradiction leads us to believe that a force-displacement curve whose secondary stable branch is shifted upwards and to the left with respect to the primary branch (as in Fig. S6a-c) cannot represent a physical system.

Nevertheless, countersnapping upon both loading and unloading could be achieved by allowing intersections between the two stable branches (Fig. S6e). Such curve respects the 'folding rules' and ensures that both countersnapping instabilities at constant displacement are associated to an energy release, under some conditions, as we will demonstrate next.

Again, we can determine the elastic energy stored in the system just before and after the countersnapping instabilities, by decomposing the integral quantity defined in Eq. (41) as a combination of areas E_i (Fig. S6f). We get the following conditions

$$0 < E_{\text{elastic}}(X_c^+) - E_{\text{elastic}}(X_r^+) \Leftrightarrow E_6 + E_4 + E_{14} + E_{15} > E_9 + E_{12} + E_{13}, \quad [44]$$

and

$$0 < E_{\text{elastic}}(X_c^-) - E_{\text{elastic}}(X_r^-) \Leftrightarrow E_7 + E_9 + E_{10} + E_{12} > E_{14} + E_{15} + E_{16}. \quad [45]$$

Conditions (44) and (45) can be satisfied simultaneously (like it is the case for the curve shown in Fig. S6e).

In conclusion, these results suggest that countersnapping upon both loading and unloading might be physically possible in a purely-elastic mechanical system. Such system would necessarily be characterized by a force-displacement curve composed of two stable branches intersecting twice; similar to the curve shown in Fig. S6e. How to design and experimentally demonstrate a mechanical system characterized by this more complex curve is an open question that we leave for future research, as it requires additional constraints compared to a system that only countersnaps upon loading.

6. Comparison of the countersnapping element with the purely-series and purely-parallel assemblies

To demonstrate that the countersnapping behavior originates from a change from a series to parallel configuration, we compare the behavior of the full assembly with the behavior of (i) the same assembly minus the stiffening building blocks, that is the purely series configuration, and (ii) the same assembly minus the snapping building block only, that is the purely parallel configuration. As it can be seen in Fig. S7, the behavior of the purely series assembly (up to its own critical point) approximates the primary stable branch of the full assembly, whereas the behavior of the purely parallel structure approximates the secondary stable branch. This observation supports the pivotal idea that the two stable configurations the structure can be in, and switch to, correspond to configurations predominantly behaving like series and parallel spring arrangements respectively.

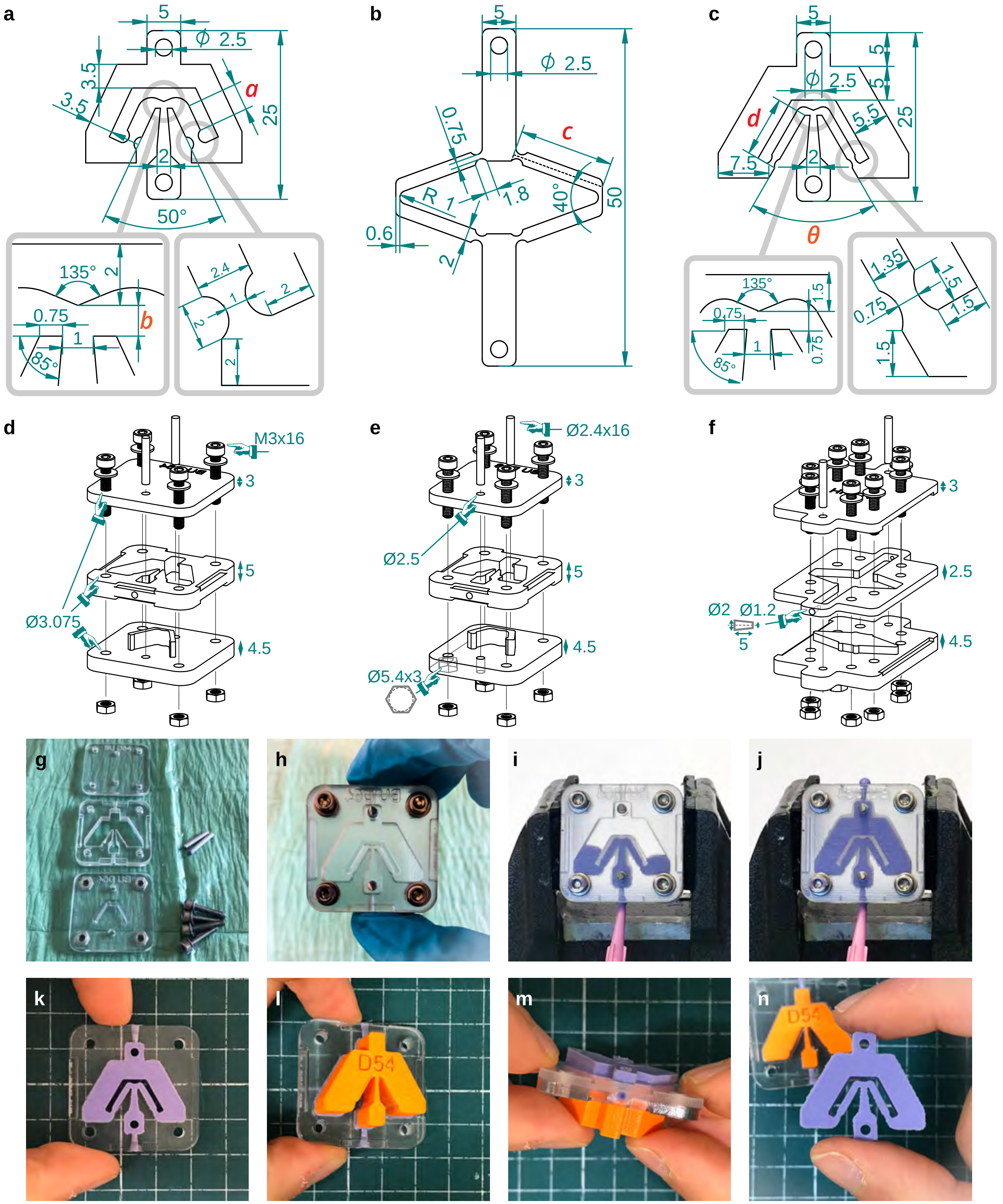

Fig. S1. Fabrication process of the building blocks. **(a-c)** Drawings of the softening (a), stiffening (b) and non-monotonic building blocks (c). Values for the design parameters a, b, c, d and θ are available in Fig. S2f. **(d-f)** Mold design and assembly for the softening (d), stiffening (e) and non-monotonic building blocks (f). **(g-n)** Mold preparation, injection procedure and demolding process for a non-monotonic building block.

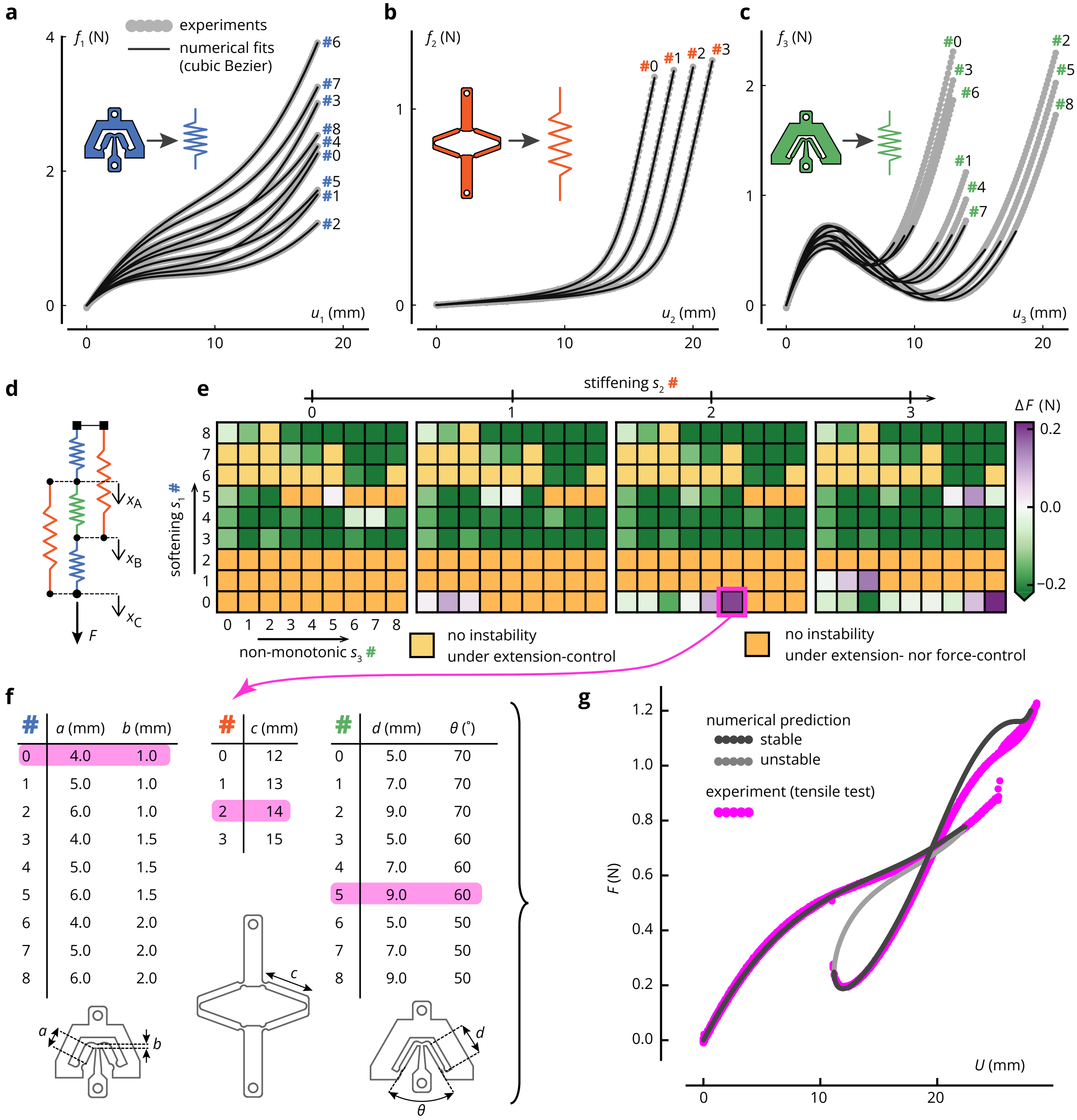

Fig. S2. Numerical approach used to guide the design of the countersnapping structure. **(a-c)** Experimental force-displacement curves and numerical fits of the softening **(a)**, stiffening **(b)** and non-monotonic **(c)** building blocks. **(d)** Nonlinear spring network. **(e)** Results of the brute-force search. Each of the 324 squares corresponds to one combination of softening, stiffening and non-monotonic building blocks. Their color indicates the sudden change in force due to the first (displacement-driven) snapping instability encountered. **(f)** Tables mapping the design id number to the set of design parameters for each type of building block. The combination of building blocks used to physically realize the countersnapping structure is highlighted in magenta. **(g)** Comparison between the force-displacement curves predicted by the numerical simulation and obtained through the tensile test experiment.

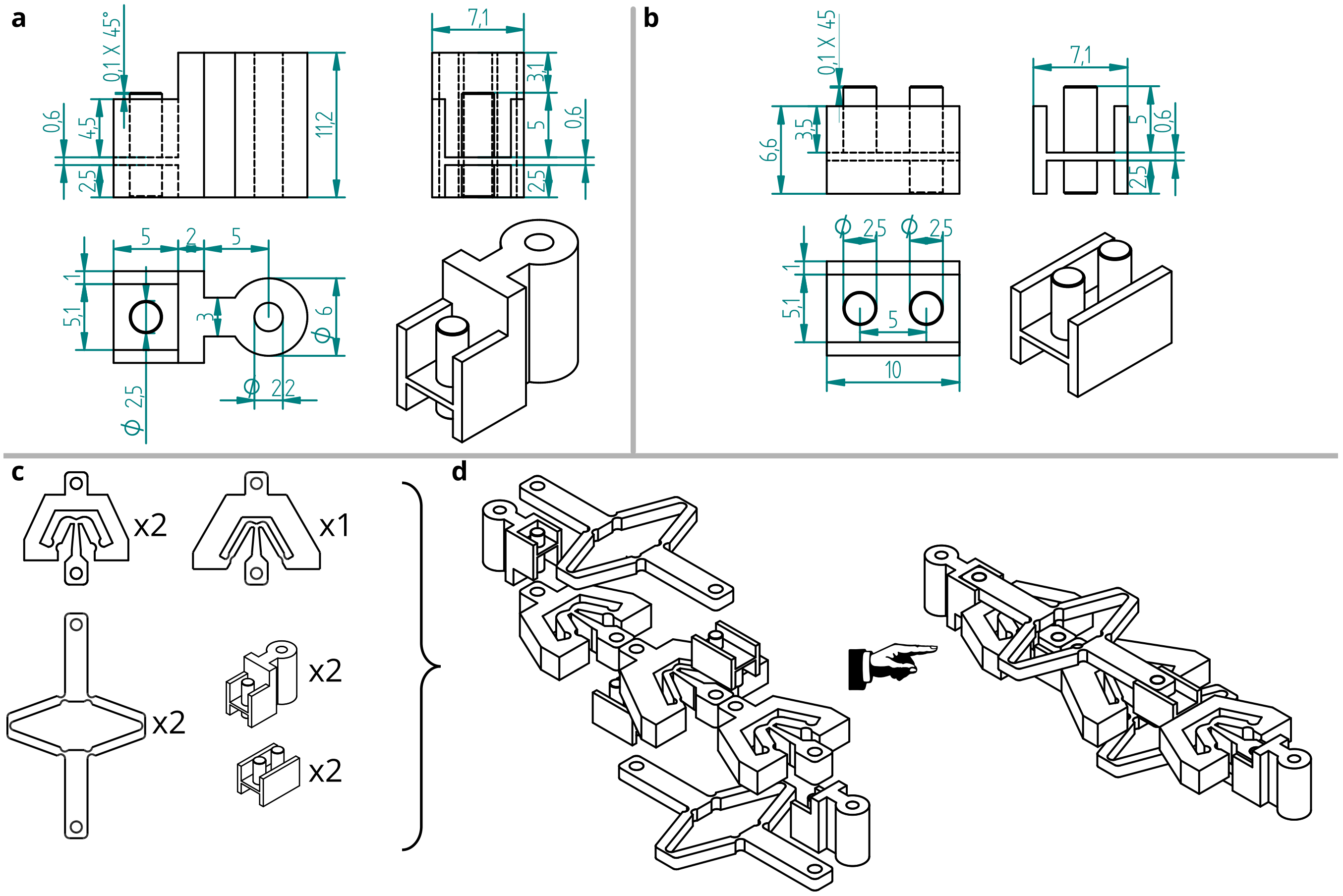

Fig. S3. Assembly of the building blocks into a countersnapping structure. **(a)** End connector drawing. **(b)** Internal connector drawing. **(c)** List of mechanical entities required for the assembly. **(d)** Assembly process.

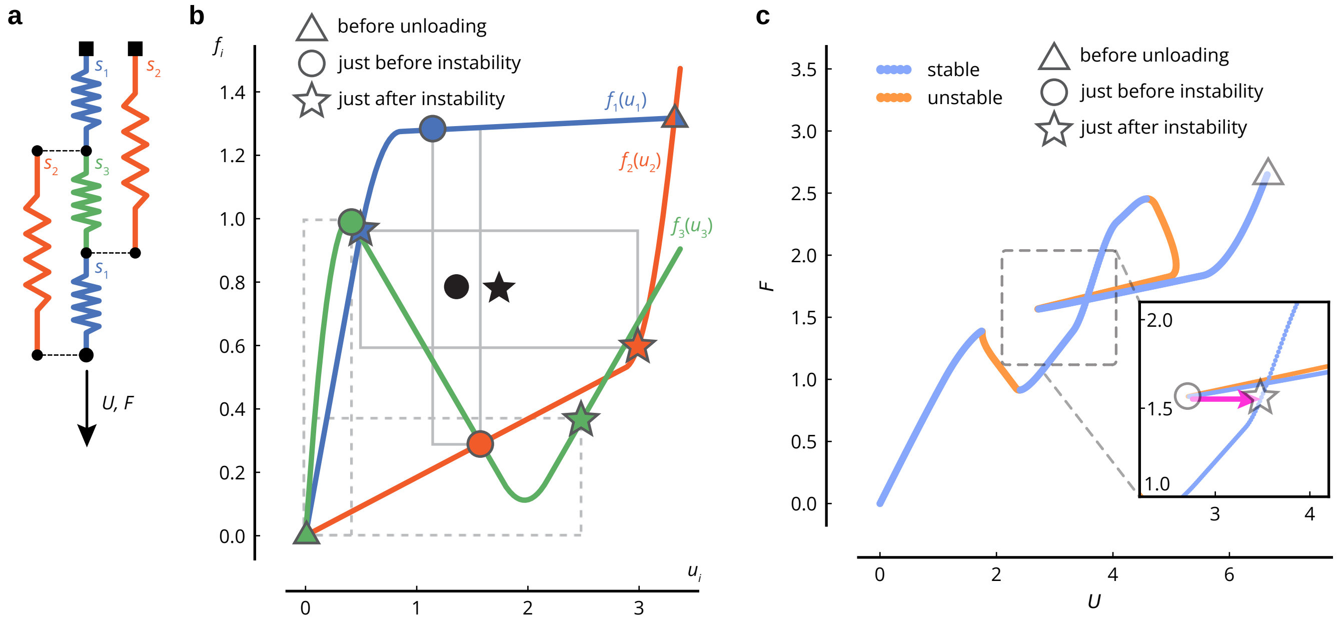

Fig. S4. Geometric construction of local force-displacement curves leading to countersnapping during unloading. **(a)** Nonlinear spring network. **(b)** Individual nonlinear force-displacement curves of each spring leading to countersnapping during unloading. **(c)** Numerically simulated force-displacement curve of the spring assembly shown in **(a)**. By unloading the structure from the equilibrium point marked by a triangle (high tension, high stretch), the first encountered instability leads to a sudden elongation as the force decreases.

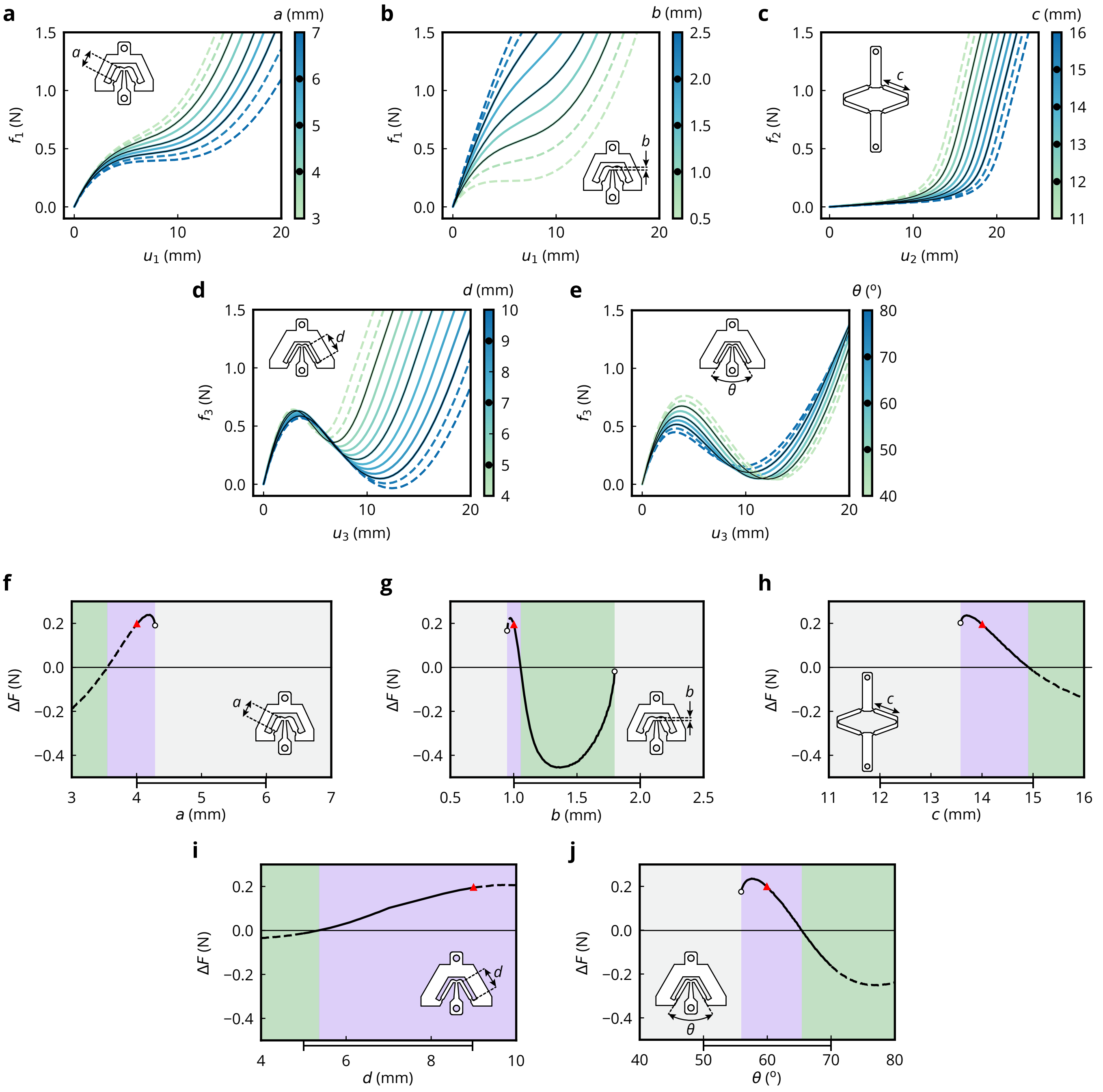

Fig. S5. Influence of the geometric parameters. **(a-e)** Estimated force-displacement curves of the individual building blocks as the geometric parameters a **(a)**, b **(b)**, c **(c)**, d **(d)**, θ **(e)** are varied. Black lines correspond to the Bezier curves fitting the experimental data, for the geometric parameter values indicated in the color bar by circular black markers. Solid (dashed) lines denote estimated force-displacement curves obtained by interpolation (extrapolation). **(f-j)** Countersnapping magnitude ΔF as a function of the geometric parameters a **(f)**, b **(g)**, c **(h)**, d **(i)**, θ **(j)**. Purple (green) backgrounds refer to regions where countersnapping, $\Delta F > 0$, (regular snapping, $\Delta F < 0$) is predicted. Gray backgrounds refer to regions where no instability occurs, ΔF is undefined. Triangular red markers indicate where the current design lies. Black solid (dashed) lines denote estimation from interpolated (extrapolated) building block force-displacement curves. Each interpolation range is depicted by a segment on the horizontal axes.

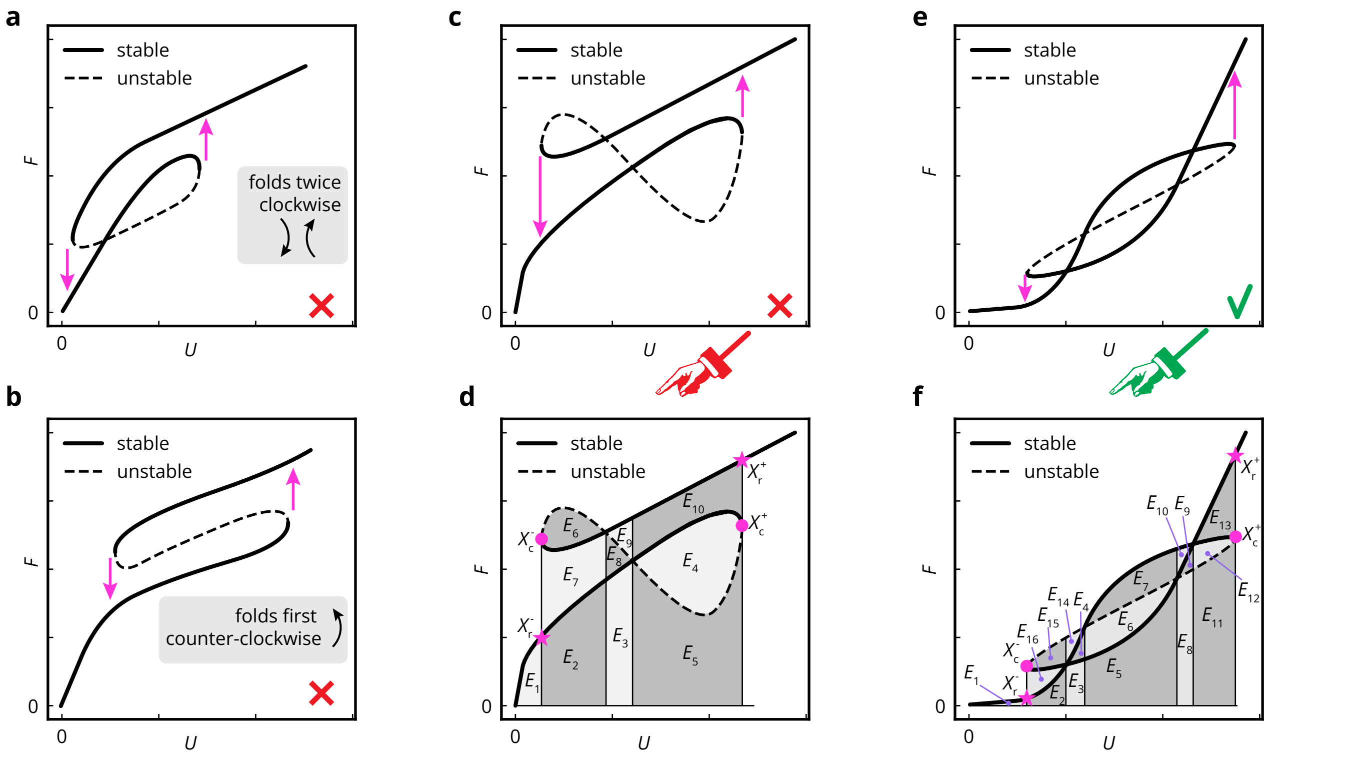

Fig. S6. Force-displacement curves that seem to lead to countersnapping upon both loading and unloading. Each curve is composed of three branches. Solid (dashed) lines denote stable (unstable) branches. Pink arrows show the snapping transitions that would occur under displacement-driven conditions. **(a)** Impossible force-displacement curve, because the two stable branches are connected by a path that folds clockwise twice. **(b)** Impossible force-displacement curve, because the two stable branches are connected a path that starts by folding counter-clockwise. **(c)** Impossible force-displacement curve, because both snapping transitions cannot be associated to an energy release. **(d)** Decomposition of the elastic energy associated to the force-displacement curve in **(c)** into areas. Pink dots (stars) refer to critical (restabilization) points. **(e)** Force-displacement curve that respects the 'folding rules' and both energy release conditions (44) and (45). **(f)** Decomposition of the elastic energy associated to the force-displacement curve in **(f)** into areas. Pink dots (stars) refer to critical (restabilization) points.

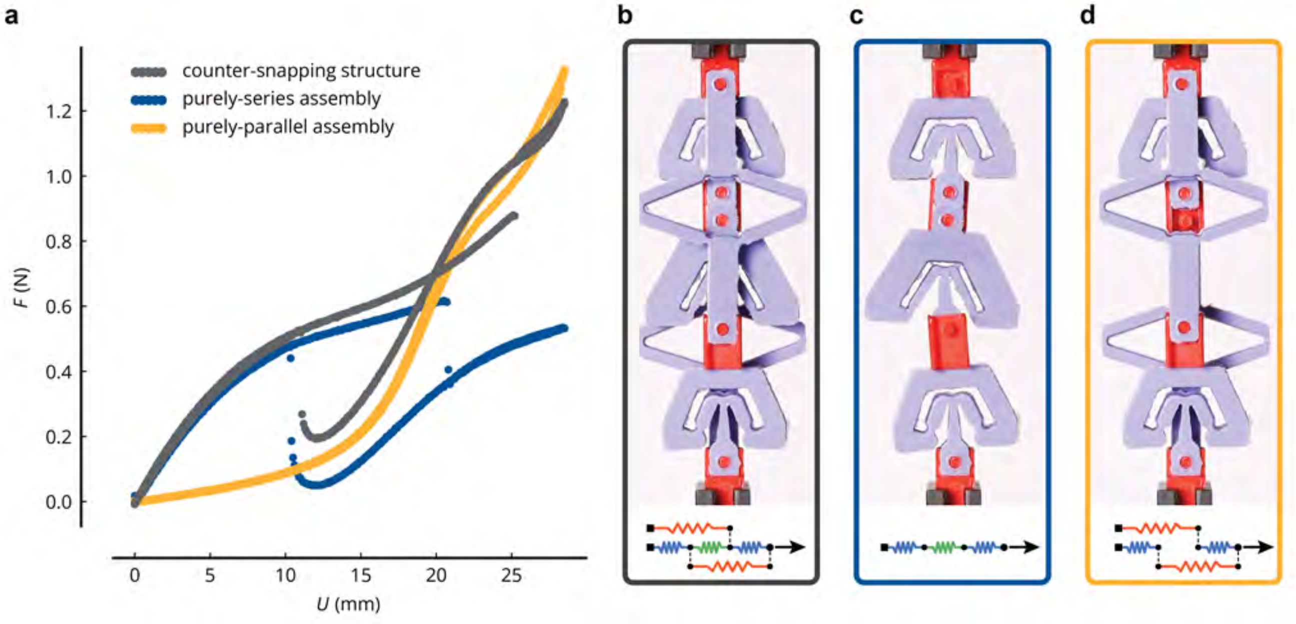

Fig. S7. Comparison of the countersnapping structure with its purely-series and purely-parallel counterparts. **(a)** Force-displacement curves of the three building-block assemblies. **(b)** Countersnapping structure. **(c)** Purely-series assembly. **(d)** Purely-parallel assembly.

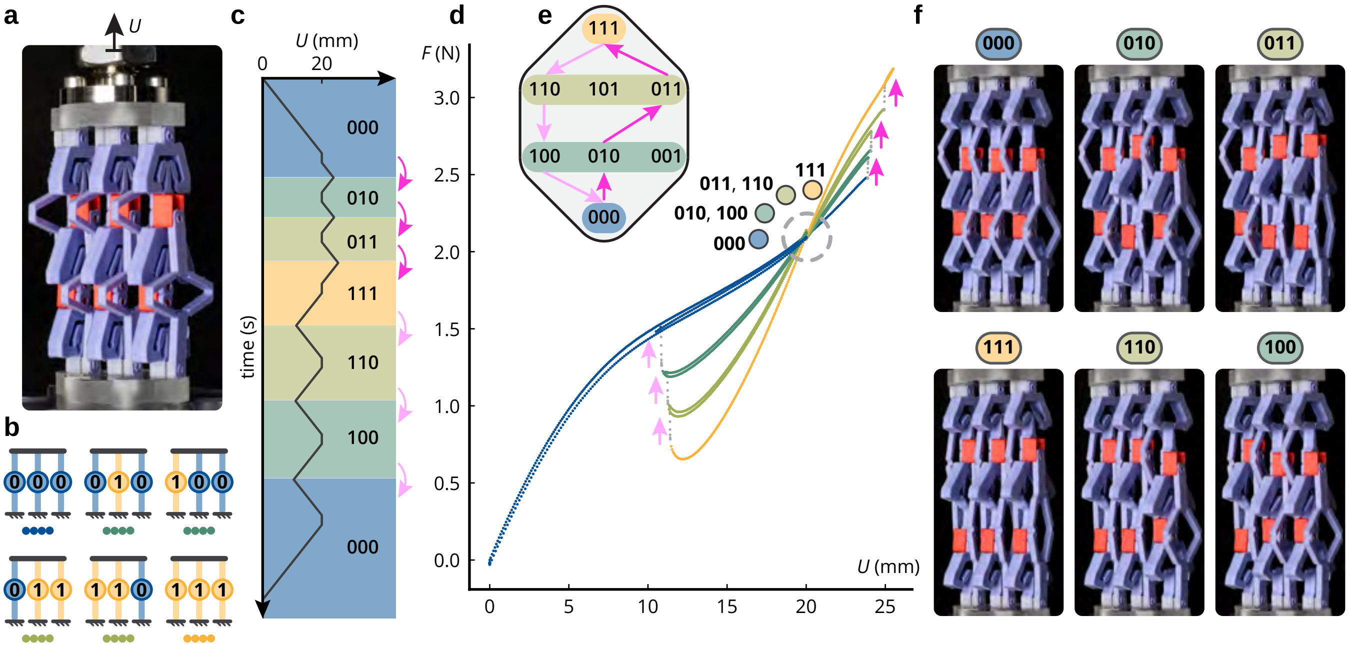

Fig. S8. Experimentally observed behavior of three countersnapping elements combined in parallel. **(a)** Three parallel countersnapping elements controlled by displacement U. **(b)** Possible collective states of the system. **(c)** The binary state of the system (background color) as function of U, where bright (light) pink arrows indicate snapping events during loading (unloading). **(d)** Corresponding force-displacement curve (colors indicating state). **(e)** Transition graph. **(f)** Snapshots of the states near the intersection point of the force-displacement curves.

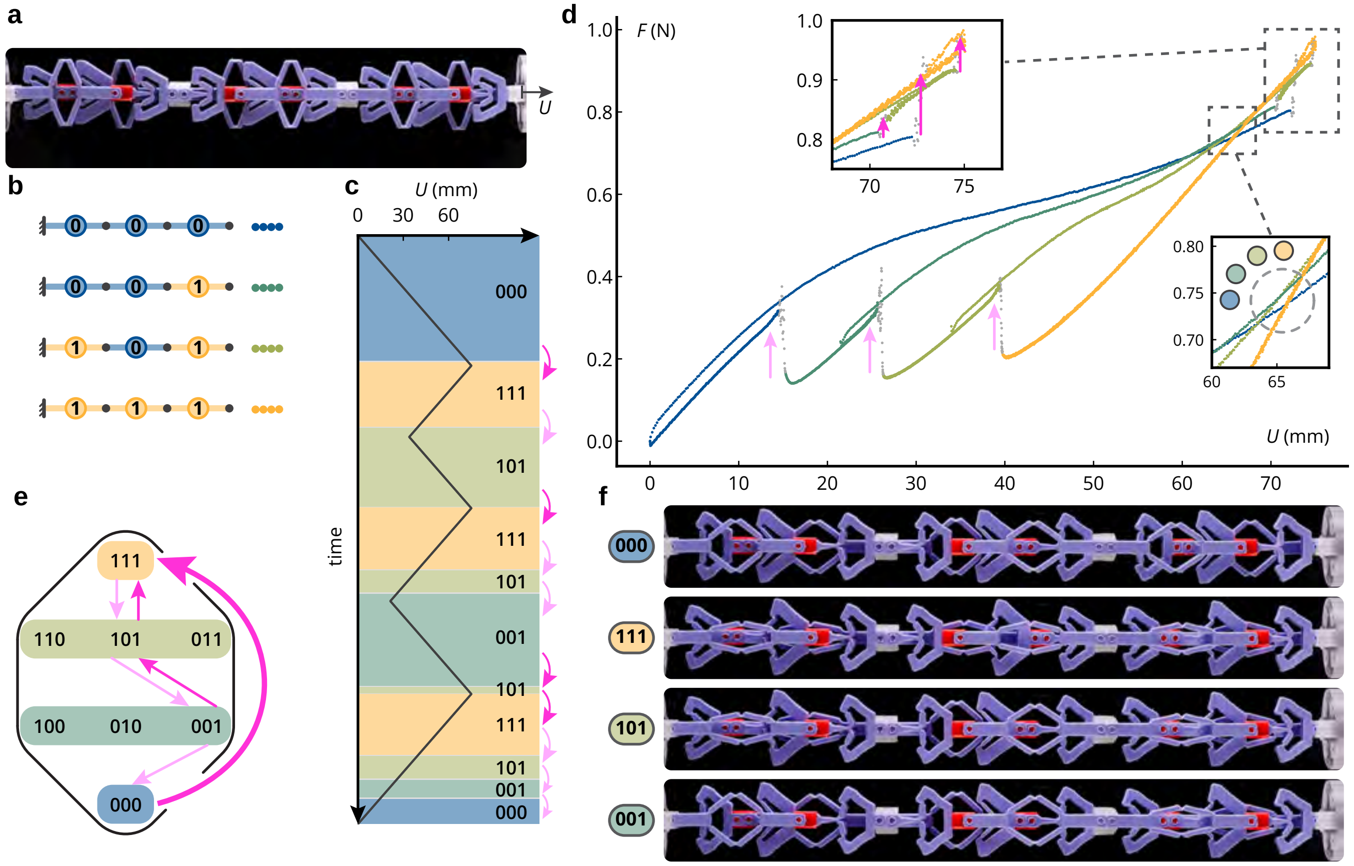

Fig. S9. Experimentally observed behavior of three countersnapping elements combined in series. **(a)** Three serially-coupled countersnapping elements controlled by displacement U. **(b)** Possible collective states of the system. **(c)** The binary state of the system (background color) as function of U, where bright (light) pink arrows indicate snapping events during loading (unloading). **(d)** Corresponding force-displacement curve (colors indicating state) **(e)** Transition graph. **(f)** Snapshots of the states near the intersection point of the force-displacement curves.

Movie S1. Tensile test of the countersnapping structure under displacement-controlled conditions. The displacement is increased then decreased, while the reaction force is measured. A sudden force jump is observed during the loading phase, and a self-intersecting force-displacement curve is obtained.

Movie S2. Tensile test of the countersnapping structure under force-controlled conditions. The tensile force is steadily increased by pouring water in a suspended cup, while the displacement is measured. A sudden contraction of the countersnapping structure (which lifts the cup) is observed while the cup is slowly filled.

Movie S3. Lifting a weight at rest on a platform and attached to the bottom of a countersnapping structure. The top of the countersnapping structure is steadily raised, while the weight elevation is measured. While raising the top of the structure, the weight, initially at rest, is suddenly lifted off the platform when the countersnapping instability is triggered.

Movie S4. Experimental comparison of the stick-slip behavior under cyclic loading of snapping and countersnapping structures. The regular snapping structure generates a back and forth actuation, while the countersnapping one generates an incremental unidirectional actuation that builds up with each cycle.

Movie S5. Experimental demonstration of programmable stiffness with unchanged equilibrium load and displacement. A countersnapping structure is preloaded to its intersection point by a suspended mass. The natural oscillation frequency of the system is measured. The frequency and the stiffness are shown to be programmable by switching between two states, which does not affect the force nor the elongation at equilibrium.

Movie S6. Experimental demonstration of self-switching stiffness upon resonance. A countersnapping structure is preloaded by a suspended mass to it intersection state then vertically vibrated from the top by a robotic arm, while the mass position is measured. When initially in the soft (stiff) state, the structure passively switches to the stiff (soft) state at the onset of resonance, which causes a reduction of the amplitude of the oscillations.

Movie S7. Collective behavior of countersnapping elements assembled in parallel and in series. By controlling the displacement to follow a certain signal over time, the state and the stiffness of the system can be programmed.

Movie S8. Avalanche transitions in serially-coupled countersnapping elements. Countersnapping elements connected in series snap together upon slowly stretching, manually poking or shaking.

References

1. MA Crisfield, A fast incremental/iterative solution procedure that handles "snap-through" in *Computational Methods in Nonlinear Structural and Solid Mechanics*, eds. AK Noor, HG McCOMB. (Pergamon), pp. 55–62 (1981).
2. M Ritto-Corrêa, D Camotim, On the arc-length and other quadratic control methods: Established, less known and new implementation procedures. *Comput. & Struct.* **86**, 1353–1368 (2008).
3. JH Maddocks, Stability and folds. *Arch. for Ration. Mech. Analysis* **99**, 301–328 (1987).
